# The Multifaceted Roles of BACH1 in Disease: Implications for Biological Functions and Therapeutic Applications

**DOI:** 10.1002/advs.202412850

**Published:** 2025-01-30

**Authors:** Xiangxiang Wei, Yunquan He, Yueyang Yu, Sichong Tang, Ruiwen Liu, Jieyu Guo, Qingjun Jiang, Xiuling Zhi, Xinhong Wang, Dan Meng

**Affiliations:** ^1^ Department of Physiology and Pathophysiology School of Basic Medical Sciences Department of Rheumatology Zhongshan Hospital Zhongshan Hospital Immunotherapy Translational Research Center Fudan University Shanghai 200032 China; ^2^ Department of Vascular & Endovascular Surgery Changzheng Hospital Naval Medical University Shanghai 200003 China

**Keywords:** BACH1, cardiovascular disease, stem cell

## Abstract

BTB domain and CNC homolog 1 (BACH1) belongs to the family of basic leucine zipper proteins and is expressed in most mammalian tissues. It can regulate its own expression and play a role in transcriptionally activating or inhibiting downstream target genes. It has a crucial role in various biological processes, such as oxidative stress, cell cycle, heme homeostasis, and immune regulation. Recent research highlights BACH1's significant regulatory roles in a series of conditions, including stem cell pluripotency maintenance and differentiation, growth, senescence, and apoptosis. BACH1 is closely associated with cardiovascular diseases and contributes to angiogenesis, atherosclerosis, restenosis, pathological cardiac hypertrophy, myocardial infarction, and ischemia/reperfusion (I/R) injury. BACH1 promotes tumor cell proliferation and metastasis by altering tumor metabolism and the epithelial‐mesenchymal transition phenotype. Moreover, BACH1 appears to show an adverse role in diseases such as neurodegenerative diseases, gastrointestinal disorders, leukemia, pulmonary fibrosis, and skin diseases. Inhibiting BACH1 may be beneficial for treating these diseases. This review summarizes the role of BACH1 and its regulatory mechanism in different cell types and diseases, proposing that precise targeted intervention of BACH1 may provide new strategies for human disease prevention and treatment.

## Graphical abstract

BACH1, a basic leucine zipper protein ubiquitously expressed in mammalian tissues, plays a critical role in the regulation of oxidative stress, cell cycle progression, heme homeostasis, and immune responses. It is implicated in cardiovascular diseases, cancer progression, neurodegenerative disorders, and many other diseases. Precise targeted intervention of BACH1 may provide new strategies for human disease prevention and treatment.

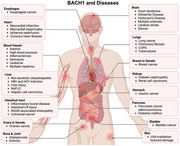

## 1 Introduction

### 1.1 Structure and Fundamental Properties of BACH1

**Figure 1:**
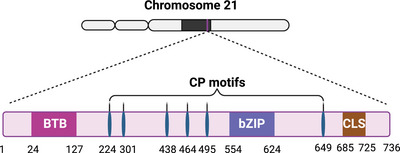
Structure and function of BACH1. BACH1, encoded on Hsa21, consists of 736 amino acids and is composed of a bZIP domain, BTB domain, cysteine–proline (CP) motifs, and cytoplasmic localization signal (CLS). The amino acid positions of cysteines are indicated by numbers. The illustration was generated using BioRender under a licensed subscription (Agreement number: TH27P0P1YR).

BACH proteins, including BACH1 and BACH2, are part of the Cap “n” Collar and basic leucine zipper (bZip) family.^[^ ^1^ ^]^ They are greatly conserved in vertebrates, particularly in functional domains (BTB and bZIP domain) and their surrounding regions.^[^ ^2^ ^]^ Yeast two‐hybrid screen identified BACH1 and BACH2 as heterodimerization partners of MAF bZIP transcription factor K (MAFK). BACH1's expression spectrum in mammalian tissues is wide, while BACH2 mainly expresses in B and T lymphocytes, natural killer (NK) cells, macrophages, and neuronal cells. BACH2 is an important regulator of innate and adaptive immunity, maintaining the function of regulatory T‐cell and crucial for B‐cell maturation. BACH2 also negatively regulates NK cell maturation and function.^[^ ^3^, ^4^ ^]^ Human BACH1 is located on chromosome 21 and consists of 736 amino acids, containing an N‐terminal BTB domain to facilitate protein interaction (**Figure** **1**). Additionally, it possesses a C‐terminal bZIP domain for DNA binding and the heterodimerization with small MAF proteins like MAFF, MAFG, and MAFK.^[^ ^1^ ^]^ Heterodimers of BACH1‐MAF bind to MAF recognition elements (MAREs, a unique DNA sequence of 5′‐TGACTCGCA‐3′) in the target gene promoters, thereby suppressing or initiating genes transcription.^[^ ^1^ ^]^ The BTB domain is crucial for multimeric protein complexes formation, especially for BACH1/MAFK heterodimers to form DNA loops. Thus, BACH1 can mediate the interaction between widely spaced cis‐regulatory elements and regulate gene expression.^[^ ^5^ ^]^ BACH1 may compete with activator protein 1 (AP‐1) factors to suppress the target genes they share.^[^ ^6^ ^]^ BACH target genes may also be affected by AP‐1 factors.^[^ ^7^ ^]^ In addition, BACH1 binds to many target DNA regions without MAFK proteins. Chromatin immunoprecipitation sequencing (ChIP‐Seq) data showed that only about 11% of the BACH1‐binding sites overlapped with MAFK‐binding sites on the whole genome in mouse embryonic fibroblasts.^[^ ^8^ ^]^ Therefore, other than MAF proteins, there may be additional mechanisms for the regulation of BACH1 target genes.

### 1.2 Epigenetic Regulation Mediated by BACH1

**Figure 2:**
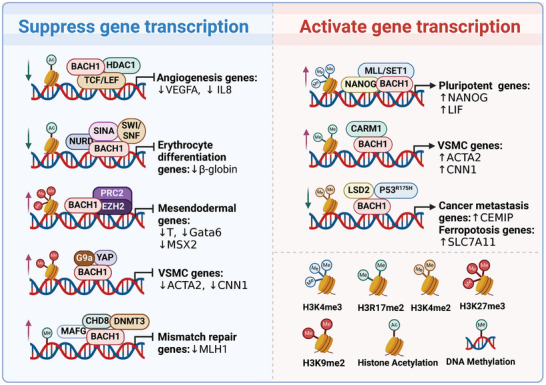
Epigenetic regulation by BACH1. BACH1 recruits partner proteins or cofactors to epigenetically transcriptionally activate or repress the expression of target genes. The illustration was generated using BioRender under a licensed subscription (Agreement number: QA27P3IE6Z).

Many studies have shown that quite a few partner proteins or cofactors bound BACH1 for the epigenetic regulation of BACH1 target gene expression (**Figure** **2**). For example, BACH1 contributes to transcriptional repression by recruiting co‐inhibitors nuclear co‐repressor 1 (NCOR1), nuclear co‐repressor 2 (NCOR2), and enzymes that modify histones such as histone deacetylase 1 (HDAC1).^[^ ^9^ ^]^ BACH1 recruits three transcriptional co‐repressor complexes (NuRD, SIN3A, and SWI/SNF) to the locus control region, which have chromatin remodeling and deacetylase activities, and thus inhibit the transcription of the β‐globin gene.^[^ ^10^ ^]^ BACH1 straightly binds to TCF4, then recruits HDAC1 to repress Wnt/β‐catenin signaling.^[^ ^11^ ^]^ BACH1 interacts with polycomb repressive complex 2 (PRC2) to suppress mesendodermal gene expression in human embryonic stem cells (hESCs) through the trimethylation of H3K27 (H3K27me3) catalyzed by enhancer of zeste homolog 2 (EZH2) in mesendodermal gene promoters.^[^ ^12^ ^]^ BACH1 suppresses the contractile phenotype of vascular smooth muscle cells (VSMCs) and recruits G9a and yes‐associated protein (YAP), which sustains the enrichment of H3K9me2 and reduces chromatin accessibility on the promoters of VSMC marker genes.^[^ ^13^ ^]^ BACH1 also induces the transcription of adhesion molecules through a combination with YAP in endothelial cells (ECs).^[^ ^14^ ^]^ BACH1 can also recruit a histone H3K4me1/2 demethylase named lysine‐specific demethylase 2 (LSD2), bind to p53^R175H^ and form BACH1‐p53^R175H^‐LSD2 complex, which modifies histone methylation status at the promoter of BACH1 targets and subsequently activates the genes transcription.^[^ ^15^ ^]^ In addition to histone modifications, BACH1 also induces DNA methylation to regulate target gene expression. The complex of BACH1 and MAFG recruits the DNA methyltransferase DNMT3B and a chromatin remodeling factor named chromodomain helicase DNA‐binding protein 8 (CHD8), resulting in hypermethylation and transcriptional silencing of tumor repressor genes.^[^ ^16^, ^17^ ^]^ BACH1 can switch from transcriptional repressors to activators, depending on the cofactors. For example, BACH1 attracts Nanog homeobox (NANOG) and mixed‐lineage leukemia/SET domain‐containing (MLL/SET1) complexes to chromatin, preserving H3K4me3 and enhancer‐promoter activity on genes related to pluripotency in mouse embryonic stem cells (mESCs),^[^ ^18^ ^]^ and recruits coactivator‐associated arginine methyltransferase 1 (CARM1) to activate the genes’ transcription during in vitro VSMC differentiation from hESCs.^[^ ^19^ ^]^ Consequently, the epigenetic regulatory roles of BACH1 might be tissue‐, cell‐type‐, or disease‐specific. Nevertheless, the precise functions of these cofactors in different contexts need further investigation. Employing multi‐omics technologies will enable a thorough analysis of the dynamic interactions between BACH1 and its cofactors.

### 1.3 Regulation of BACH1 Stabilization

**Figure 3:**
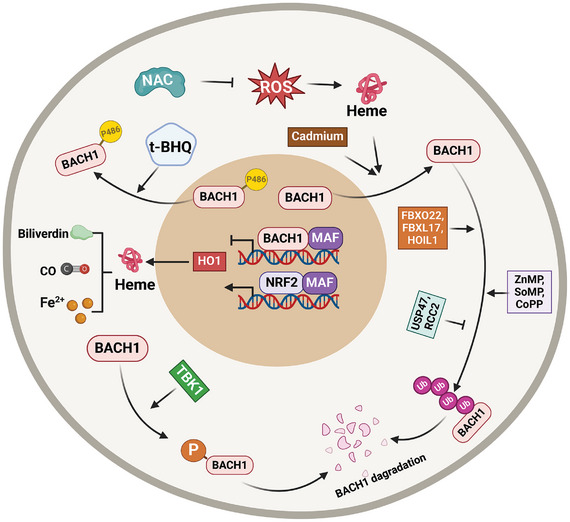
Regulation of BACH1 stabilization. The regulation of BACH1's stabilization, as well as its nuclear export and import, is controlled by multiple signals or regulators via post‐translational mechanisms, including phosphorylation and ubiquitination‐dependent degradation. The illustration was generated using BioRender under a licensed subscription (Agreement number: EH27P3ILN8).

At high heme concentration, heme directly binds to BACH1 via six cysteine‐proline motifs located in an intrinsically disordered region, and heme binding induces a global conformational change in BACH1's heme‐binding region,^[^ ^20^ ^‐^ ^22^ ^]^ thus inactivating BACH1 DNA‐binding activity,^[^ ^23^ ^]^ and facilitating BACH1's nuclear export and degradation. Consequently, heme oxygenase 1 (HO‐1) expression is induced. HO‐1 subsequently degrades heme into the biologically active metabolites carbon monoxide (CO), biliverdin, and ferrous iron (Fe^2+^).^[^ ^24^, ^25^ ^]^ Thus, the increased HO‑1 stabilizes BACH1 by degrading heme. BACH1, in turn, suppresses the expression level of HO‐1. Therefore, BACH1/HO‐1/heme are mutually regulated by each other, this negative feedback ensures the homeostasis of heme.^[^ ^26^ ^]^ The nuclear export signals of BACH1 triggered by heme depend on chromosome region maintenance 1 (CRM1).^[^ ^27^, ^28^ ^]^ When BACH1 is exported to the cytoplasm, it colocalizes with intracellular hyaluronic acid‐binding protein (IHABP) [also known as hyaluronan‐mediated motility receptor (HMMR)] and forms a fibrous structure on microtubules.^[^ ^29^ ^]^ During mitosis, BACH1, IHABP, and CRM1 stabilize mitotic spindle orientation. The cell cycle‐specific phosphorylation of BACH1 in mitosis acts as the switch that changes its function from transcription regulation to mitosis.^[^ ^30^ ^]^ Heme triggers BACH1 degradation through the ubiquitin‐proteasome pathway by promoting its interaction with the ubiquitin E3 ligase adaptor proteins F‐box protein 22 (FBXO22, a cullin‐RING ligase 1 (CRL1) substrate receptor), and the activated NRF2 induces HO‐1 expression, thus represses the FBXO22‐dependent degradation of BACH1 in lung cancer cells.^[^ ^31^, ^32^ ^]^ The activated NRF2 restores BACH1 levels, which might be a negative feedback regulation for NRF2 pathway. In addition, BACH1 is also polyubiquitinated by F‐box and leucine‐rich repeat protein 17 (FBXL17)^[^ ^33^, ^34^ ^]^ and heme‐oxidized IRP2 ubiquitin ligase 1 (HOIL‐1).^[^ ^35^ ^]^ The inhibitors of proteasome or neddylation suppress heme‐induced BACH1 degradation.^[^ ^35^ ^]^ Certain non‐heme metalloporphyrins include zinc mesoporphyrin (ZnMP), tin mesoporphyrin (SnMP), and cobalt protoporphyrin (CoPP), but the metallic ions and the free porphyrins don't downregulate the BACH1 protein by increasing proteasomal degradation.^[^ ^36^ ^]^ The nuclear export of BACH1, triggered by Cadmium, is dependent on the function of the extracellular signal‐regulated protein kinase (ERK1/2) within the mitogen‐activated protein kinase pathway.^[^ ^37^ ^]^ The antioxidant *tert*‐butylhydroquinone (t‐BHQ) triggers a swift nuclear export of BACH1, permitting NRF2 to activate the expression of antioxidant genes. Additionally, the phosphorylation of BACH1 at tyrosine 486 by antioxidants is essential for its export from the nucleus.^[^ ^38^ ^]^ In contrast, antioxidants, such as vitamins C and E and N‐acetylcysteine (NAC), prevent BACH1 degradation and inhibit the release of free heme by lowering ROS levels,^[^ ^39^ ^]^ since ROS stimulates free heme release through the oxidization of heme‐containing proteins.^[^ ^40^ ^]^ Therefore, therapeutic use of antioxidants must consider their complex impact on BACH1. Recently, BACH1 has been shown to be degraded by TANK binding kinase 1 (TBK1), a serine/threonine kinase. TBK1 facilitates the degradation of BACH1 via both phosphorylation‐dependent and ‐independent mechanisms independently of FBXO22 or heme.^[^ ^41^ ^]^ In contrast, ubiquitin‐specific peptidase 47 (USP47) directly binds to BACH1, deubiquitinates and stabilizes BACH1 for the promotion of Warburg effect and the progression of non‐small cell lung cancer.^[^ ^42^ ^]^ The mitotic regulator RCC2 (regulator of chromatin condensation 2) stabilizes BACH1 protein through participating in C‐terminus ubiquitination.^[^ ^43^ ^]^ Thus, BACH1 degradation relies on nuclear export, ubiquitination, and deubiquitination, highlighting the need for precise regulation of its stability to adapt to different cellular conditions (**Figure** **3**).

### 1.4 Upstream Regulators of BACH1

**Figure 4:**
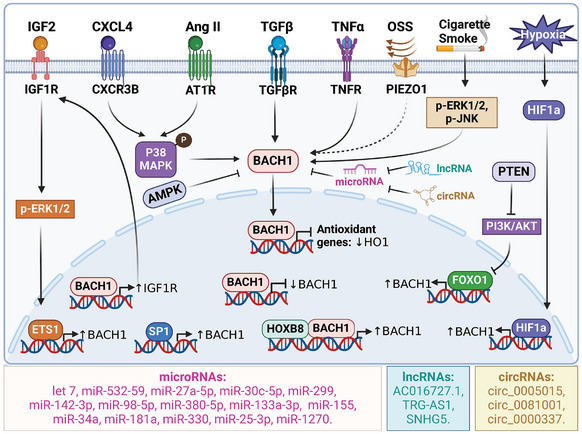
Upstream regulators of BACH1. BACH1 regulation by transcriptional and post‐transcriptional mechanisms. Various Cytokines or stimuli enhance or suppress BACH1 expression through distinct signaling pathways. IGF2 facilitates BACH1 upregulation via the ERK1/2/ETS1 pathway. Moreover, BACH1 targets the IGF1R, interacting with IGF2, to establish a positive feedback loop, which continuously enhances the expression of BACH1. PNET induces BACH1 transcription through up‐regulation of FOXO1 and inhibition of PI3K‐AKT. BACH1 mRNA can be directly negatively regulated by miRNAs or indirectly regulated by LncRNAs and circRNAs. The illustration was generated using BioRender under a licensed subscription (Agreement number: ZO27P0M4A6).

Multiple signaling pathways participate in regulating the expression of BACH1 (**Figure** **4**). The transcription factor specificity protein 1 (SP1) has the ability to attach to the BACH1 gene's promoter region, thereby controlling its fundamental level of expression.^[^ ^44^ ^]^ BACH1 was found to possess autoregulatory mechanisms. BACH1 can hinder its own transcription via binding to its own promoter region, suggesting that it operates as a transcriptional suppressor with a regulatory mechanism of negative feedback.^[^ ^45^ ^]^ In contrast, BACH1 also activates its own transcription by binding with HOXB8 in colorectal cancer cells.^[^ ^46^ ^]^ Additionally, the p38/MAPK(mitogen‐activated protein kinases) pathway activated by C‐X‐C chemokine receptor type 3‐B (CXCR3‐B) can cause the movement of BACH1 into the nucleus and decrease HO‐1′s level in breast cancer cells.^[^ ^47^ ^]^ ERK1/2 and JNK also play a role in cigarette smoke‐induced BACH1 expression in human macrophages.^[^ ^48^ ^]^ Angiotensin II (Ang II) promotes the expression of BACH1 via p38/MAPK signaling pathway in cardiomyocytes.^[^ ^49^ ^]^ Insulin‐like growth factor 2 (IGF2) upregulates BACH1 via ERK1/2/ETS1 signaling pathway in hepatocellular carcinoma.^[^ ^50^ ^]^ Transforming growth factor β (TGF‐β) also upregulates BACH1 and MAFK expression, and then suppresses HO‐1 expression in mouse mammary gland epithelial cells.^[^ ^51^ ^]^ BACH1 expression and nuclear localization are also increased in endothelial cells exposed to disturbed flow compared with laminar shear stress.^[^ ^14^, ^52^ ^]^ BACH1 was induced both under 5% O_2_ and 1% O_2_ in endothelial cells.^[^ ^11^, ^53^ ^]^ The expression of BACH1 increased in a hypoxia‐inducible factor 1 alpha (HIF1α)‐dependent and independent manners upon hypoxia.^[^ ^54^ ^]^ Moreover, inhibition of BACH1 expression also decreased hypoxia‐induced HIF‐1α expression, indicating the BACH1/HIF‐1α signaling loop formed in a hypoxic environment.^[^ ^55^ ^]^ Phosphate and tensin homology deleted on chromosome ten (PTEN) promotes forkhead box O1 (FOXO1) recruitment to the BACH1 promoters, leading to the transcription of BACH1, and increased PTEN activity or inhibition of PI3K‐AKT also up‐regulates BACH1 at the posttranscriptional levels in cholangiocarcinoma cells.^[^ ^56^, ^57^ ^]^ AMPK has been shown to negatively regulate BACH1 mRNA expression, leading to the transactivation of a subset of NRF2 target genes.^[^ ^58^ ^]^ Besides, many microRNAs (miRs) are the key regulators for BACH1 mRNA expression. For example, miR‐532‐5p, miR‐27a‐5p, miR‐30c‐5p, miR‐299, miR‐142‐3p, miR‐98‐5p, miR‐380‐5p, miR‐133a‐3p, miR‐155, miR‐34a, miR‐181a, miR‐330, miR‐25‐3p, and miR‐1270 have been shown to inhibit BACH1 mRNA level.^[^ ^59^, ^60^, ^61^, ^62^, ^63^, ^64^, ^65^, ^66^, ^67^, ^68^, ^69^, ^70^, ^71^, ^72^, ^73^ ^]^ Long non‐coding RNA (lncRNA) AC016727.1 and TRG‑AS1 increase BACH1 expression by inhibiting miR‐98‐5p and miR‑4500 in non‐small cell lung cancer cells and hepatocellular carcinoma respectively,^[^ ^55^, ^74^ ^]^ while Let7 inhibits BACH1 expression in breast cancer cells, lung alveolar type 2(AT2) cells and palate cells.^[^ ^75^, ^76^, ^77^ ^]^ LncRNA SNHG5 enhances BACH1 levels by directly interacting with miR‐299 in breast cancer cells.^[^ ^62^ ^]^ Certain circular RNAs, such as circ_0005015, circ_0081001, and circ_0000337, have the ability to enhance cancer progression by upregulating BACH1 via the mechanism of microRNA sponging as well.^[^ ^78^, ^79^, ^80^ ^]^ Nevertheless, additional investigation is required to comprehend the mechanisms through which BACH1 expression is induced, including low oxygen induction, oxidative stress‐induced inflammatory factors, hormones and cytokines, and other potential pathways.

### 1.5 BACH1 in Oxidative Stress and Senescence

BACH1 is essential for the regulation of oxidative stress under pathological conditions.^[^ ^81^, ^82^ ^]^ Under oxidative stress conditions, BACH1 is regulated by ROS, which inactivates BACH1 through oxidizing its cysteine residue,^[^ ^83^ ^]^ leading to the up‐regulation of antioxidant genes like HO‐1, NQO1, GCLC, GCLM, and SLC7A11.^[^ ^84^ ^]^ Mice lacking BACH1 exhibit chronically elevated levels of HO‐1, leading to enhanced resilience against oxidative stress‐related disorders such as colitis, lung injury, fatty liver disease, cardiovascular diseases, pulmonary fibrosis, and nervous system dysfunctions.^[^ ^85^, ^86^, ^87^, ^88^, ^89^, ^90^, ^91^, ^92^, ^93^ ^]^ Decreased BACH1 expression/activity diminishes apoptosis of pancreatic β‐cells,^[^ ^94^ ^]^ shields keratinocytes against UV damage,^[^ ^95^ ^]^ mitigates intervertebral disc degeneration,^[^ ^96^ ^]^ and hinders apoptosis in trophoblast cells of pre‐eclampsia.^[^ ^66^ ^]^ BACH1 overexpression enhances ROS production, resulting in increased apoptosis and decreased angiogenesis.^[^ ^97^, ^98^ ^]^ Elevated BACH1 levels increase oxidative stress‐induced DNA damage associated with triptolide‐induced nephrotoxicity.^[^ ^99^ ^]^ BACH1 knockdown represses lipopolysaccharide (LPS)‐triggered oxidative stress, inflammatory factors, and ferroptosis‐related genes in human bronchial epithelial cells, which is linked to NRF2/HO‐1 signaling.^[^ ^100^ ^]^ Consequently, the modulation of BACH1 expression is integral to cellular responses to oxidative stress, the maintenance of heme homeostasis, and the defense against pathological conditions. Moreover, it may offer potential therapeutic targets for various diseases, including hepatic, pulmonary, and cardiovascular disorders.

**Figure 5:**
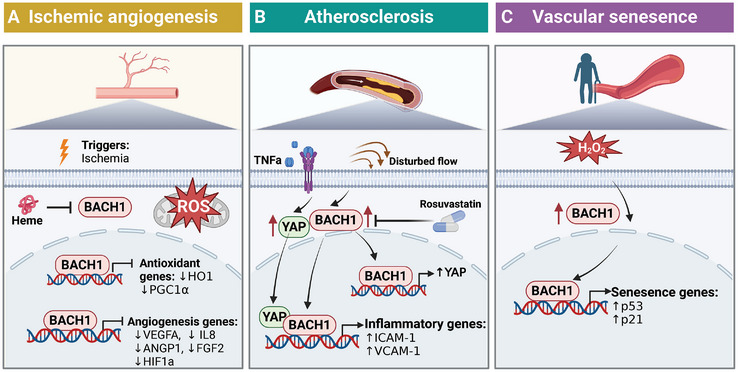
The role of BACH1 in endothelial cells. BACH1 attenuates angiogenesis in ischemic conditions by downregulating pro‐angiogenic factors and antioxidant genes. Conversely, BACH1 exacerbates atherosclerosis through the modulation of endothelial cell inflammation and contributes to the promotion of vascular senescence. The illustration was generated using BioRender under a licensed subscription (Agreement number: UX27P3JAPU).

In the aging process, ROS accumulation, dysfunctional mitochondria, and DNA damage are significantly involved. BACH1 and NRF2 are strongly related to aging and age‐related diseases.^[^ ^101^, ^102^ ^]^ NRF2 acts as a key regulator in the life‐prolonging signaling pathway via the regulation of antioxidant expression,^[^ ^103^, ^104^ ^]^ NRF2 deletion can cause a wide range of aging‐related transcriptomic changes in age‐related diseases.^[^ ^105^ ^]^ With aging, BACH1 expression is increased in mouse embryonic fibroblast cells (MEF) and aortas of aged monkey and mouse.^[^ ^106^, ^107^, ^108^ ^]^ In an accelerated aging mouse model, the accumulation of BACH1 in the nucleus increases under H_2_O_2_ treatment in embryonic fibroblasts.^[^ ^106^ ^]^ There are a number of target genes for BACH1 that are associated with senescence. BACH1 inhibition stimulates NRF2 signaling and induces antioxidant genes expression, which inhibits the occurrence of cellular senescence under oxidative stress, and thus prevents mitochondrial ROS production.^[^ ^97^, ^109^, ^110^ ^]^ It seems that BACH1 can be a potential target for anti‐aging intervention. Gene regulatory network analysis shows that BACH1 is the main regulator of age‐related genes in mouse endothelial cells. BACH1‐knockdown downregulates the expression of senescence‐associated secretory phenotype (SASP) components, alleviating the senescence caused by oxidative stress in endothelial cells. BACH1 binds to P21 gene enhancer and upregulates it in endothelial cells. Protective effect of BACH1 inhibition was possibly through downregulating P21 and P53, leading to cell cycle arrest prevention. The findings indicate that BACH1 is a crucial transcription factor in human endothelial cell senescence (**Figure** **5**C).^[^ ^107^ ^]^ However, previous study has shown that BACH1 inhibits cellular senescence through repressing P53 in mouse embryonic fibroblasts. BACH1 recruits HDAC1 to some target genes required for senescence of P53, leading to a decreased expression.^[^ ^9^ ^]^ Differences in senescence between endothelial cells and fibroblasts remain unclear, but this may be due to the different cell types, cell states, cell responses to oxidative stress, or experimental conditions.

## 2 Functions of BACH1 in Cardiovascular Diseases

### 2.1 BACH1 and Ischemic Angiogenesis

The process of angiogenesis is intricate and comprises various stages, such as activation, movement, multiplication, and the creation of structures resembling tubes by endothelial cells.^[^ ^111^, ^112^, ^113^, ^114^ ^]^ HIF‐1 induction induced by hypoxia or dimethyloxaloylglycine (DMOG) increases BACH1 expression and attenuates NRF2‐dependent HO‐1 induction in human endothelial cells.^[^ ^115^ ^]^ HO‐1 expression has been linked to increases in the migration and tube formation of ECs and angiogenesis.^[^ ^116^ ^]^ Our previous research has shown that BACH1 is a negative regulator of post‐ischemic neovascularization in adult mice. BACH1 inhibits the binding of β‐catenin/TCF4 and recruits HDAC1 to the promoter of TCF4 target genes, leading to the reduced expression of vascular endothelial growth factor (VEGF), interleukin‐8 (IL‐8), and various pro‐angiogenic factors.^[^ ^11^, ^97^ ^]^ Additional research has verified that BACH1 has a direct interaction with TCF4, which is facilitated by the N‐terminal BTB domain residues 81–89 of BACH1 and the N‐terminal domain of TCF4. Absence of the BTB domain in BACH1 results in the inability to bind and activate HDAC1.^[^ ^117^ ^]^ Hence, this BTB domain actively engages in diverse molecular interactions, enhancing the anti‐angiogenic function of BACH1, and presenting itself as a promising target for drug development in angiogenesis therapy.^[^ ^117^ ^]^ Blood flow recovery following hindlimb ischemia was significantly enhanced in BACH1/Apolipoprotein E (ApoE) double‐knockout (KO) mice compared to ApoE KO mice. The upregulation of HO‐1 and peroxisome proliferator‐activated receptor γ co‐activator‐1α (PGC‐1α) caused by ablation of BACH1 may repress the ROS generation, and increased angiopoietin 1 (ANGP‐1) and fibroblast growth factor 2 (FGF2) in ECs may facilitate the angiogenesis after hindlimb ischemia.^[^ ^118^ ^]^ BACH1 also inhibits the expression of ANGP‐1 in pericytes, and less secreted ANGP‐1 leads to decreased in vitro EC network formation.^[^ ^59^ ^]^ Similarly, heme inhibits BACH1 expression, promotes VEGF expression, and relieves the impaired angiogenesis response in human microvascular endothelial cells induced by hyperoxia.^[^ ^119^ ^]^ In addition, BACH1 over‐expression inhibits ECs proliferation and increases mitochondrial ROS production to promote cell‐cycle arrest and apoptosis.^[^ ^97^ ^]^ Therefore, BACH1 inhibits angiogenesis through at least two mechanisms, one that is mediated by decreased angiogenesis‐related genes (including HO‐1, VEGF, IL‐8, ANGP‐1, and FGF2), and another that involves increased ROS production and apoptosis in ECs (Figure 5A).

### 2.2 BACH1 in Atherosclerosis and Restenosis

The initiation and advancement of atherosclerosis are significantly influenced by vascular inflammation and endothelial dysfunction.^[^ ^120^, ^121^, ^122^, ^123^, ^124^ ^]^ The genome‐wide association study (GWAS) study has shown that BACH1 resides in the proximity of a risk locus (rs2832227) for coronary artery disease (CAD).^[^ ^125^ ^]^ Our recent research has shown that this risk variant was related to BACH1 gene expression in patients’ carotid plaques. BACH1 is highly expressed in endothelial cells of atherosclerotic plaques in both humans and mice, particularly in the aortic arch, where oscillatory shear stress is elevated. BACH1's upregulation is also seen in the carotid plaques of individuals with cerebrovascular symptoms, suggesting a potential role in atherosclerosis progression. Disturbed flow leads to vascular inflammation and atherosclerosis. BACH1 is activated by oscillatory shear stress (OSS) and increases adhesion molecules and monocyte‐endothelial adhesion. BACH1 plays a vital role as a mechano‐transducer in regulating endothelial inflammation in reaction to local blood flow dynamics, thereby mediating atheroprone phenotypes caused by disturbed flow and contributing to the development of atherosclerotic lesions. BACH1 is also upregulated by LPS, TNF‐α, IL‐1β, ox‐LDL, hypoxia, and oxidative stress in ECs.^[^ ^14^, ^126^ ^]^ Upon stimulation by OSS or TNF‐α, both BACH1 and YAP are activated and transported into the nucleus in ECs. BACH1 increases YAP expression via binding to its promoter and forms a complex with YAP to enhance the transcription of adhesion molecules. Additionally, the global deletion of BACH1 in mice has been shown to reduce atherosclerosis progression through the upregulation of HO‐1 expression, a known protective mechanism against oxidative stress.^[^ ^127^ ^]^ Therefore, there are at least two mechanisms for BACH1 deficiency to alleviate atherosclerotic lesion formation: firstly, it can have an anti‐inflammatory impact by suppressing YAP activation in ECs, and secondly, it can produce anti‐oxidative effects dependent on HO‐1. Importantly, rosuvastatin suppresses the expression of BACH1 via let‐7a, downregulates BACH1 expression in vascular endothelium and relieves vascular inflammation. In addition, MAFF‐BACH1 heterodimers binding at the MAF recognition element in the low‐density lipoprotein receptor (LDLR) promoter significantly reduces LDLR expression in the human hepatocytes, indicating MAFF‐BACH1 may be associated with lipid and lipoprotein metabolism in the liver.^[^ ^126^ ^]^ Therefore, BACH1 plays a complex role in atherosclerosis, involving multiple aspects such as oxidative stress, inflammatory response, endothelial function, and lipid metabolism. intervening in BACH1 may offer new therapeutic targets for cardiovascular diseases and metabolic diseases (Figure 5B).

**Figure 6:**
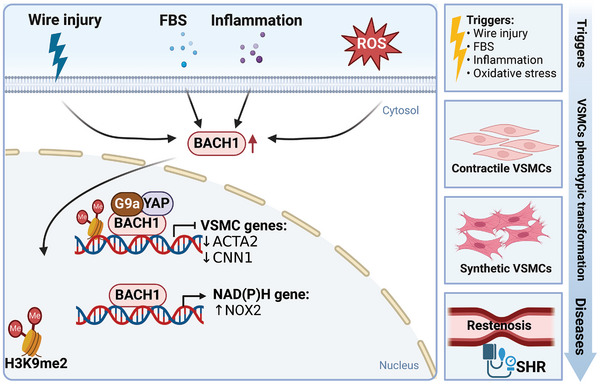
The role of BACH1 in VSMCs. In VSMCs, BACH1 expression is induced by triggers like ROS, inflammation, FBS, and wire injury. BACH1 suppresses the expression of smooth muscle markers and antioxidant genes while enhancing the expression of NAD(P)H. Additionally, BACH1 facilitates the synthetic transformation of vascular smooth muscle cells, contributing to restenosis and the development of spontaneous hypertension in rat models (SHR). The illustration was generated using BioRender under a licensed subscription (Agreement number: TS27P3JTR0).

Restenosis after vascular surgery is mainly caused by intimal hyperplasia, which occurs upon vessel injury and involves inflammation, VSMCs dedifferentiation, migration, proliferation, and matrix secretion into the intima.^[^ ^128^, ^129^, ^130^, ^131^ ^]^ The genetic variant rs73193808 associated with CAD has allele‐specific enhancer activity in human aortic smooth muscle cells (HASMCs),^[^ ^132^ ^]^ and this variant is closest to the BACH1 gene. We have identified that the region with rs73193808 is also accessible to BACH1 in VSMCs, indicating a possible link between them in atherosclerotic lesions. Increased BACH1 expression is observed in VSMCs in the mouse model of wire‐injured femoral arteries. Additionally, BACH1 is induced and translocated to the nucleus in HASMCs after serum stimulation. The global deletion of BACH1 in mice has been demonstrated to reduce the growth and neointimal formation of VSMCs in an HO‐1‐independent way.^[^ ^133^ ^]^ VSMC phenotype switching is vital in intimal hyperplasia.^[^ ^134^ ^]^ We have shown that VSMC‐specific deletion of BACH1 prevents the conversion of VSMC from contractile to synthetic phenotype, reduces VSMC proliferation, and decreases neointimal hyperplasia in wire‐injured femoral arteries of mice. BACH1 plays a crucial role in suppressing the contractile phenotype of VSMCs by enriching H3K9me2 through the recruitment of G9a and YAP, resulting in decreased chromatin accessibility of VSMC marker gene promoters like ACTA2 and CNN1. Accordingly, a recent study also found that BACH1 and BACH2 are the candidate key regulators in the phenotypic modulation of VSMC in healthy individuals and Marfan syndrome patients by analyzing of single‐nucleus multi‐omic and spatial transcriptomic sequencing data.^[^ ^135^ ^]^ In addition, the knockdown of BACH1 also suppresses the production of ROS and NAD(P)H oxidase subunit NOX2 expression and decreases cell migration in VSMCs from spontaneously hypertensive rats (SHRs).^[^ ^136^ ^]^ These findings offer ponderable insights into the regulatory function of BACH1 for the phenotypic transformation of VSMC and maintenance of vascular homeostasis.^[^ ^13^ ^]^ (**Figure** **6**) Endothelial regeneration after injury reduces restenosis risk in coronary artery interventions.^[^ ^137^ ^]^ Considering the increased EC proliferation and migration by loss of BACH1,^[^ ^11^ ^]^ BACH1 deficiency potentially reduces intimal hyperplasia through maintaining VSMC phenotype and promoting endothelial regeneration.

### 2.3 BACH1 and Myocardial Hypertrophy

**Figure 7:**
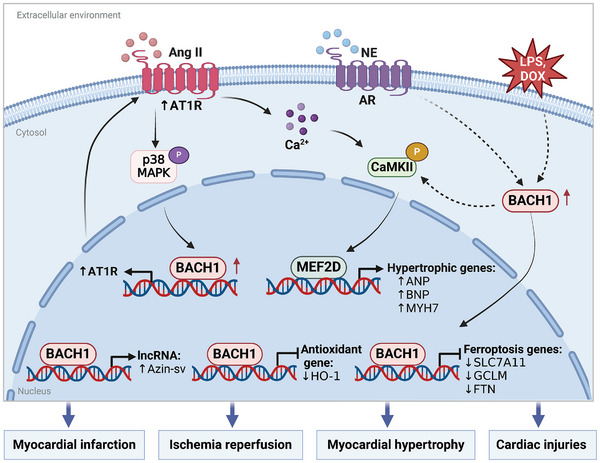
The role of BACH1 in CMs. Several factors, including Angiotensin II (Ang II), norepinephrine (NE), doxorubicin (DOX), and lipopolysaccharide (LPS), have been shown to upregulate BACH1 expression in cardiomyocytes (CMs). BACH1 plays a pivotal role in the advancement of pathological cardiac hypertrophy, ischemia/reperfusion injury, myocardial infarction, and various other cardiac pathologies. It exerts its effects through multiple signaling pathways, notably by enhancing the expression of hypertrophic genes and suppressing the expression of genes associated with antioxidant defense and ferroptosis. The illustration was generated using BioRender under a licensed subscription (Agreement number: MC27P3K106).

Pathological hypertrophy results in malignant cardiac remodeling and heart failure, characterized by fibrosis, capillary rarefaction, elevated pro‐inflammatory cytokines, and cellular dysfunction.^[^ ^138^, ^139^, ^140^, ^141^ ^]^ Previous studies have indicated that mice lacking BACH1 had reduced left ventricular hypertrophy and fibrosis caused by transverse aortic constriction (TAC) by increasing HO‐1 expression.^[^ ^142^ ^]^ The upregulation of BACH1 in cardiac tissues is observed in both human pathological cardiac hypertrophy and heart failure.^[^ ^49^ ^]^ Our study demonstrated that targeted loss of BACH1 in mouse cardiac tissue mitigates cardiac hypertrophy caused by Ang II and pressure overload, reduces cardiac fibrosis, and maintains cardiac function. Additionally, in an in vitro cardiac hypertrophy model, BACH1 overexpression stimulates the growth of cardiomyocytes treated with Ang II and norepinephrine while silencing BACH1 attenuates this effect. Ang II‐activated p38/MAPK facilitates BACH1 translocation into the nucleus in cardiomyocytes, and promotes its recruitment to the angiotensin II type 1 receptor (AT1R) gene promoter, therefore activating AT1R transcription. The inhibition of BACH1 results in a reduction of Ang II‐ and norepinephrine‐induced CaMKII signaling pathway activation and hypertrophic gene expression in neonatal rat cardiomyocytes.^[^ ^49^ ^]^ BACH1‐mediated enhancement of cardiac hypertrophy in response to Ang II is found to be modulated, at least partially, by AT1R expression both in vivo and in vitro. It indicates that BACH1 is a significant regulatory factor in pathological cardiac hypertrophy through its control of AT1R expression and the Ca^2+^/CaMKII pathway. Furthermore, a significant number of patients exhibit intolerance to angiotensin‐converting enzyme inhibitors (ACEIs) and angiotensin II receptor blockers (ARBs). Therefore, silencing BACH1 might serve as a potential treatment by targeting AT1R in individuals who are unable to benefit from the inhibition of this pathway. Targeting BACH1 shows great promise as an innovative approach to prevent and treat pathological cardiac hypertrophy and heart failure (**Figure** **7**).

### 2.4 BACH1 and I/R Injury or Myocardial Infarction

Ischemia/reperfusion (I/R) injury continues to pose a challenging issue in cases of acute ischemic heart disease subsequent to interventional therapy.^[^ ^143^, ^144^ ^]^ The occurrence of oxidative stress is vital in I/R injury as it initiates a series of events that lead to tissue damage.^[^ ^145^ ^]^ BACH1 regulates the cellular redox state and affects damage caused by oxidative stress during I/R by controlling the expression of HO‐1. Previous research has demonstrated that the elimination of the BACH1 gene results in safeguarding the heart from damage caused by I/R in mice. The use of zinc‐protoporphyrin, an inhibitor for HO activity, eliminates the ability of BACH1 deficiency to reduce the occurrence of infarction. This suggests that the decrease in the size of the infarct by ablation of BACH1 was mediated by the activity of HO‐1.^[^ ^88^ ^]^ Additionally, the cardioprotective ability of BACH1 disruption is also associated with STAT3 activation and inhibition of p38/MAPK signaling and apoptosis in myocardial tissues.^[^ ^88^ ^]^ Treatment with Na_2_S leads to Erk phosphorylation, resulting in BACH1 nuclear export and NRF2 signaling activation, ultimately reducing myocardial ischemia‐reperfusion injury in db/db mice.^[^ ^146^ ^]^ Similarly, miR‐30c‐5p has been shown to improve myocardial I/R injury by activating NRF2 and inhibiting BACH1.^[^ ^61^ ^]^ Additionally, BACH1 enhances the expression of the lncRNA‐AZIN2 splice variant (AZIN2‐sv) by binding to its promoter, thereby inhibiting angiogenesis and myocardial regeneration after the myocardial infarction (MI).^[^ ^147^ ^]^ Multiple researches have shown that ferroptosis is involved in the heart's ischemia‐reperfusion damage. BACH1 has been found to repress the expression of ferritin, ferroportin, and HO‐1, thereby facilitating ferroptosis and aggravating acute myocardial infarction. Knockout of BACH1 in mice has shown increased resistance to acute myocardial infarction due to reduced levels of ferroptosis.^[^ ^148^ ^]^ Ferroptotic cell death is promoted by catecholamine stimulation of cardiomyocytes via BACH1‐HO‐1 and GPX4 signaling.^[^ ^149^ ^]^ BACH1 knockout in mice protected against doxorubicin (DOX)‐induced ferroptosis and cardiomyopathy through increasing the expression of HO‐1.^[^ ^150^ ^]^ Sepsis is a worldwide health issue causing widespread inflammation, oxidative stress, and multi‐organ failure, especially affecting the heart.^[^ ^151^, ^152^, ^153^, ^154^ ^]^ Ferroptosis in septic cardiomyopathy could be mitigated by modulating m6A methylation of BACH1 with FTO, an essential regulator of m6A methylation.^[^ ^155^ ^]^ Consequently, targeting BACH1 could be a promising therapeutic strategy for acute myocardial infarction, I/R, and sepsis‐induced cardiac injuries in the future (Figure 7).

## 3 BACH1 in Stem Cells and Cell Differentiation

### 3.1 The Regulation of Pluripotency or Stemness in Stem Cells

Pluripotency is defined as the capacity of cells to differentiate into multiple cell lineages within an organism.^[^ ^156^ ^]^ The same core pluripotency factors, OCT4 (POU class 5 homeobox 1, POU5F1, alternatively called OCT4), NANOG (Nanog homeobox, NANOG), and SOX2 (SRY‐box transcription factor 2, SOX2) are key factors in maintaining pluripotency by activating genes and each other in mouse embryonic stem cells (mESCs) and in human embryonic stem cells (hESCs).^[^ ^157^ ^]^ Mice that have homozygous deletions of all BACH1‐coding exons are sublethal, suggesting BACH1 is crucial during mouse embryonic development. We have shown that BACH1 is strongly expressed and colocalized with OCT4 in mouse blastocysts’ inner cell mass. BACH1 preserves the pluripotency of hESCs by stabilizing stemness factors (including NANOG, SOX2, and OCT4), which is mediated by recruiting the deubiquitinating factor USP7. BACH1 deletion in hESCs activates the mesendodermal gene expression and mesendodermal differentiation. The BACH1's function to silence mesendodermal gene expression is due to the PRC2‐mediated H3K27me3 deposition at genes’ promoters, and the inhibition of Wnt3 and Nodal signaling. Thus, BACH1 is a determining factor for cell destiny determination and lineage specification in hESCs.^[^ ^12^ ^]^ In addition to the stabilization of pluripotency factors, BACH1 also functions as a mediator protein in chromatin loops by facilitating the recruitment of key pluripotent factor NANOG and histone‐modifying enzymes MLL/SET1 complexes in mESCs. This process serves to regulate the promoter‐enhancer activity, particularly those involving super‐enhancers, thereby enhancing the transcription of pluripotency‐regulating genes and ultimately supporting the maintenance of pluripotency. The interactions and maintenance of pluripotency necessitate the presence of both BTB and bZIP domains in BACH1 in mESCs. Unlike in hESCs, BACH1 does not colocalize with H3K27me3 at the genes’ promoters in mESCs. The underlying causes of the differences between human and mouse embryonic stem cells (ESCs) remain unidentified. A plausible explanation is the existence of distinct pluripotent states in human and mouse ESCs, specifically primed versus naïve states. Alternatively, these differences may depend on the involvement of diverse transcriptional regulators and epigenetic pathways in maintaining pluripotency.

Increasing evidence indicates that cancer stem cells (CSCs) function crucially in the initiation of tumors and are the primary drivers of carcinoma metastasis.^[^ ^158^, ^159^, ^160^ ^]^ The biological function of CSCs is regulated by pluripotent factors such as OCT4, SOX2, and NANOG, as is widely recognized.^[^ ^161^ ^]^ Similar to the role in ESCs, BACH1 also induces lung cancer stem cell phenotypes by upregulating the expression of CD44. Inhibition of BACH1 is able to suppress tumor‐initiating CSC markers (OCT4, SOX2, NANOG, and CD44), tumor‐sphere formation, and CSC growth and metastasis in the xenograft model of mice.^[^ ^162^ ^]^ BACH1 mediated the upregulation of mitochondrial ROS and CSC‐marker expression stimulated by chronic intermittent hypoxia.^[^ ^163^ ^]^ Accordingly, BACH1 also elevates the mRNA levels of stemness‐associated genes (including CD133, and CD44), and contributes to maintaining the cancer stemness of hepatocellular carcinoma cells. Moreover, thioredoxin promoted the stemness of hepatocellular carcinoma cells by interacting with BACH1, stabilizing BACH1 expression by inhibiting its ubiquitination, and facilitated hepatocellular carcinoma metastasis both in vitro and in vivo.^[^ ^164^ ^]^ Thus, BACH1 may act as a central hub, bringing together NANOG, SOX2, and OCT4 to stabilize these factors, and meanwhile, it increases the transcription of CD44 and CD133, ultimately contributing to the enhancement of cancer stem cell‐like properties.

### 3.2 BACH1 and Erythroid Differentiation

**Figure 8:**
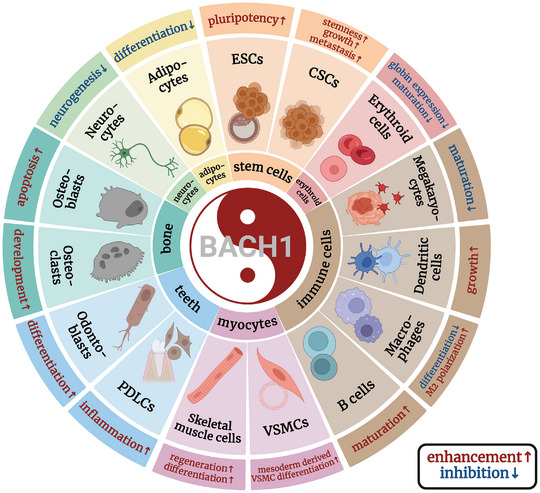
BACH1 in stem cells and cell differentiation. BACH1 maintains the self‐renewal and pluripotency of embryonic stem cells and also promotes the proliferation and metastasis of cancer stem cells. It has different roles of promoting and inhibiting in the differentiation and development of various tissues and cells. The illustration was generated using BioRender under a licensed subscription (Agreement number: AU27P3K7U0).

BACH1 is involved in the differentiation of a variety of cells (**Figure** **8** and **Table** **1**). The interaction between heme and BACH1 is crucial in regulating globin gene expression. The human globin gene cluster, which spans 70 kb, consists of five globin genes (ϵ, γG, γA, δ, and β) that are governed by the microlocus control region (µLCR). BACH1 forms heterodimers with small MAF proteins and recruits transcriptional corepressor complexes [nucleosome remodeling and deacetylase (NuRD), switch‐insensitive 3a (SIN3A), or switch/sucrose nonfermentable (SWI/SNF)] to the µLCR of the β‐globin gene, thus inhibiting the β‐globin gene expression.^[^ ^10^ ^]^ Heme binds to BACH1 and blocks the interaction between BACH1 and MARE regions in the µLCR, thus promoting globin gene expression.^[^ ^165^, ^166^ ^]^ GATA1, a crucial regulator of erythropoiesis, initiates the expression of genes required for red blood cell maturation, like globin genes and enzymes involved in heme biosynthesis. GATA‐1 promotes globin gene transcription when heme biosynthesis functions normally. However, in erythroid cells lacking heme, BACH1 accumulates and inhibits globin's transcription mediated by GATA‐1.^[^ ^167^, ^168^, ^169^ ^]^ BACH1 and BACH2 have been demonstrated to inhibit myeloid genes and promote erythroid genes by competing with C/EBPβ. Depletion of BACH1 or BACH2 in human CD34^+^ hematopoietic stem and progenitor cells (HSPCs) leads to impaired erythroid differentiation in vitro. Therefore, BACH1 and BACH2 facilitate erythroid commitment via repressing the myeloid program under steady conditions, playing a physiological role of “balancers” in the erythro‐myeloid lineage decision. Reduced activity of BACH transcription factors may be implicated in the pathogenesis of anemia of inflammation and myelodysplastic syndrome.^[^ ^170^ ^]^ BACH1/BACH2 regulates erythroid differentiation and globin gene expression through a complex network, playing critical roles in both physiological and pathological states. Future studies may clarify their molecular roles and explore clinical targeting opportunities.

**Table 1:** Effect of BACH1 in stem cells and cell differentiation.

Cell type		Co‐factor	Target genes	Functions	Refs.
Stem cells	hESCs	USP7	Stemness genes (Nanog, Sox2 and Oct4)	Maintains pluripotency by stabilizing Nanog, Sox2 and Oct4	[12]
		PRC2 complexes	Mesendodermal genes (T, Msx2, Gata4/6)	Suppresses mesendodermal gene expression through PRC2‐mediated H3K27me3 deposition at genes’ promotors Inhibiting Wnt3 and Nodal signaling	
	mESCs	NANOG, MLL/SET1 complexes	Stemness‐related genes (Nanog, Zfp42, Lif)	Enhances activity of super enhancers by facilitating chromatin loops, supports pluripotency maintenance	[18]
	CSCs	NANOG, SOX2, OCT4	CSC markers (CD133, CD44)	Induces stemness‐associated genes, promotes CSC phenotype, tumor‐sphere formation and metastasis	[158, 159, 160, 161, 162, 163, 164]
Erythroid cells		NuRD, SIN3A, SWI/SNF	β‐globin gene,	Forming heterodimers with small Maf proteins, recruiting transcriptional corepressor complexes (NuRD, SIN3A, SWI/SNF) to the microlocus control region of the β‐globin gene, and inhibiting the β‐globin gene exprssion	[10]
		—–	GATA1	BACH1 accumulating and inhibiting globin's transcription mediated by GATA‐1 in erythroid cells lacking heme	[165, 166, 167, 168, 169]
		—–	Myeloid genes	BACH1/2 accelerate erythroid commitment via repressing the myeloid program at steady state, playing a physiological role of ‘balancers’ in the erythro‐myeloid lineage decision	[170]
Immune cells	APCs	—–	SPI‐C	Repressing SPI‐C, thus suppressing the differentiation of red pulp macrophages from monocytes	[171, 172, 173, 174]
	B cells	—–	Myeloid genes	BACH1/2 inhibit myeloid genes in common lymphoid progenitors (CLPs), leading to the enhancement of B cell maturation	[175]
	Megakaryocytes	—–	MARE‐dependent genes (Thromboxane synthase)	Impaired megakaryocyte maturation and thrombocytopenia occur when BACH1 is overexpressed	[177]
Myocytes	VSMCs	CARM1	VSMC marker genes (ACTA2, CNN1)	Facilitating the recruitment of CARM1 to the promoters of VSMC marker genes, thereby enhancing their transcription through the augmentation of H3R17me2 modification	[19]
	C2C12	—–	Smad2, Smad3, Foxo1	Inhibition of BACH1 leads to decreased cell proliferation, formation of myotubes, and expression of myogenin potentially by the up‐regulation of Smad2, Smad3, and FoxO1, which suppresses muscle cell differentiation	[193]
Adipocytes		—–	PPARγ	Suppressing the expression of PPARγ and PPARγ‐dependent adipocyte differentiation and adipogenesis	[8, 194, 195]
Osteoblasts		—–	NRF2‐mediated antioxidant enzymes	Translocating into nucleus and attenuating NRF2‐mediated antioxidant enzymes, thus increasing intracellular reactive oxygen species signaling and osteoclastogenesis in mice	[196, 197, 198, 199, 200, 201, 202, 203, 204, 205, 206, 207]
Odontoblasts		—–	Mineralization markers	Promoting the proliferation and odontoblastic differentiation of hDPSCs	[208, 209, 210, 211, 212]
PDLCs		EZH2	RUNX2, BMP6	Hindering the expression of osteoblastic genes Silencing BACH1 shields PDLCs from inflammatory harm during periodontal bone regeneration	[213, 214, 215]

### 3.3 BACH1 and Immune Cell Differentiation

BACH serves as a crucial regulatory factor for innate and adaptive immune systems, such as influencing the differentiation of B and T cell lineages, CD4^+^ regulatory T cells, and macrophages.^[^ ^6^ ^]^ BACH1 is crucial in the production of antigen‐presenting cells (APCs), including macrophages and dendritic cells, that are vital for both immune systems, as well as maintaining self‐immune tolerance. Mice without BACH1 exhibit hindered APCs growth, resulting in compromised T‐cell reactions and limited defense against experimental autoimmune encephalomyelitis, a condition marked by inflammation and harm to the central nervous system.^[^ ^171^ ^]^ In mouse tissue macrophages, gene expression profiles and transcriptional regulatory pathways determine their identity as well as their diversity. BACH1 was one of the transcriptional regulators responsible for controlling these essential genes associated with macrophages.^[^ ^172^ ^]^ BACH1 has been demonstrated to repress the transcription factor SPI‐C in a manner independent of MAFK or NRF2. SPI‐C functions vitally for macrophage formation in the spleen and bone marrow. Thus, BACH1 suppresses the transformation of monocytes into red pulp macrophages, while an increased level of heme inactivates BACH1, resulting in red pulp macrophages differentiation.^[^ ^173^, ^174^ ^]^ BACH1, together with BACH2, are essential in the development of B cells by inhibiting myeloid genes in common lymphoid progenitors (CLPs), enhancing B cell maturation, and inhibiting myeloid maturation in CLPs.^[^ ^175^ ^]^ BACH2 competes with AP‑1 transcription factors and inhibits AP‑1‑driven gene expression, preventing improper T cell receptor (TCR)‐driven induction of effector programs in cells intended for a memory cell destiny. Therefore, BACH2 enhances B cell proliferation and the differentiation of memory cells.^[^ ^6^ ^]^ BACH1 and BACH2 have been implicated in the regulation of both commitment and on‐demand hematopoiesis and are suppressed upon infectious and inflammatory conditions.^[^ ^176^ ^]^ BACH1 also controls the development of megakaryocytes, which have the responsibility of generating platelets. Impaired megakaryocyte maturation and thrombocytopenia occur when BACH1 is overexpressed in transgenic mice, regulated by the GATA1 promoter. This could be due to the inhibition of various p45 targeted genes, including thromboxane synthase, by BACH.^[^ ^177^ ^]^ Tumor‐associated macrophages (TAMs) are essential in tumor progression, which can be classified into M1 and M2 types. M1 macrophages promote beneficial immune response via pro‐inflammatory cytokines, while M2 macrophages regulate immunity and predominantly support tumor growth.^[^ ^178^ ^]^ Studies have revealed that LINC00665 works in conjunction with BACH1 to trigger WNT1 activation, thereby influencing the M2 polarization of tumor‐associated macrophages in gastric cancer.^[^ ^179^ ^]^ Conversely, there are contradictory findings on the role of BACH1 in macrophage polarization. A study reported an unconventional phenotype of LPS‐mediated BACH1‐defective macrophages marked by both pro‐inflammatory traits (enhanced inflammasome activation) as well as anti‐inflammatory (high expression levels of HO‐1 and M2 markers) attributes. This indicates a middle stage in the macrophage activation range.^[^ ^180^, ^181^ ^]^ Additionally, genetic variations in the BACH2 locus have been linked with a higher risk of autoimmune and allergic diseases,^[^ ^182^, ^183^ ^]^ future work is needed to elucidate the specific roles of BACH proteins in human immunity.

### 3.4 BACH1 and VSMC or Myoblast Differentiation

Lineage‐specific differentiation of stem cells into VSMCs is essential for regenerative medicine.^[^ ^184^, ^185^, ^186^, ^187^ ^]^ BACH1 functions in regulating mesodermal cell differentiation into VSMCs in hESCs. Increased BACH1 expression correlates with VSMC marker expression during hESC differentiation. Deleting BACH1 hinders VSMC marker gene expression and reduces hESC differentiation efficiency. Overexpressing BACH1 after mesoderm induction boosts VSMC marker expression, but BACH1 overexpression on Days 1–3 inhibits mesoderm differentiation in hESCs. BACH1 facilitates the recruitment of CARM1 to the promoters of VSMC marker genes, thereby enhancing their transcription through the augmentation of H3R17me2 modification, thus playing a role in the differentiation of VSMCs following mesoderm induction.^[^ ^19^ ^]^ The upregulation of VSMC marker genes in cultured mature human aortic smooth muscle cells following BACH1 silencing,^[^ ^13^ ^]^ suggests the varying effects of BACH1 on mesoderm‐derived cell differentiation and mature VSMCs. The diverse impacts of BACH1 may be attributed to differences in extracellular and intracellular environments, cofactors, and cellular contexts.

Muscle development is the process by which precursor cells differentiate into mature muscle fibers.^[^ ^188^ ^]^ This complex process involves a series of cellular and molecular events that are tightly regulated to ensure proper muscle formation.^[^ ^189^ ^]^ Analysis of bovine skeletal muscle development using transcription and open chromatin single‐cell sequencing predicted the specific expression of transcription factors (TFs), such as BACH1, which are associated with muscle development.^[^ ^190^, ^191^ ^]^ BACH1‐deficient mice have higher HO‐1 expression level, which relieves tissue injuries. However, BACH1 global knockout mice exhibit damaged muscle regeneration, disorganized macrophage phenotype transition, and transcriptional deregulation of crucial repair‐related and inflammatory genes in the cardiotoxin‐induced skeletal muscle injury mouse model. BACH1 directly modulates numerous critical inflammatory and repair‐related genes in muscle‐derived macrophages following cardiotoxin injury, including IGF1, SLC40A1, IL‐6, IL‐10, GDF3, PPARG, DUSP1, and CEBPB, potentially elucidating the delayed muscle regeneration phenotype observed in BACH1 KO mice. The expression of HO‐1 may participate in transitioning macrophages from an inflammatory phenotype to a reparative phenotype.^[^ ^192^ ^]^ Another report has shown that inhibition of BACH1 in C2C12 myoblast cells leads to decreased cell proliferation, formation of myotubes, and expression of myogenin potentially by the up‐regulation of SMAD2, SMAD3, and FOXO1, which suppresses the differentiation of muscle cells.^[^ ^193^ ^]^ These findings suggest BACH1's role in muscle regeneration and differentiation, but its potential involvement in other non‐muscle cells aiding muscle cell differentiation or regeneration cannot be ruled out, as a global deletion of mice was utilized. Additional research employing skeletal muscle‐ or macrophage‐specific deficiencies of BACH1 will be required to investigate these matters further.

### 3.5 BACH1 and Adipocyte Differentiation

BACH1 also participates in controlling essential genes necessary for the differentiation of adipocytes. BACH1 exhibited significant induction throughout the process of adipogenesis.^[^ ^194^ ^]^ The expression of peroxisome proliferator‐activated receptor (PPAR)γ and PPARγ‐dependent adipocyte differentiation is suppressed by BACH1 in primary mouse embryonic fibroblasts, suggesting that BACH1 is an inhibitory regulator of adipocyte differentiation and adipogenesis.^[^ ^8^ ^]^ A prior study found that progesterone receptor membrane component 2 (PGRMC2) is necessary for transporting labile heme into the nucleus. Deleting PGMRC2 in brown fat leads to a decrease in labile heme levels in the nucleus and stabilizes BACH1, which influences mitochondrial bioenergetics. Therefore, altered gene expression leads to severe mitochondrial abnormalities, preventing adipose‐specific PGRMC2‐null mice from inducing adaptive thermogenesis and making them more susceptible to pronounced metabolic decline when fed a high‐fat diet.^[^ ^195^ ^]^ Hence, BACH1 is crucial in adipocyte differentiation and adipocyte function.

### 3.6 BACH1 in Osteoblast and Osteoclast or Osteoclastogenesis

Fluoride is a trace element necessary for accurate bone and tooth development.^[^ ^196^ ^]^ Excessive systemic exposure to fluoride (drinking water and food) may result in fluorosis.^[^ ^197^ ^]^ Whole genome bisulfite sequencing (WGBS), combined analysis of promoter DNA hypermethylation was performed in fluoride‐exposed human osteosarcoma cells (HOS) and identified epigenetic changes in the BACH1 gene, essential for skeletal morphogenesis/development, ossification, and osteoblast development.^[^ ^197^ ^]^ Another study suggests that the inhibition of BACH1 attenuates oxidative stress‐induced osteoblast apoptosis and impairment via the amplification of NRF2/ARE signaling, highlighting the BACH1/NRF2/ARE regulatory axis in the development of osteoporosis.^[^ ^198^ ^]^ The equilibrium between osteoblast‐mediated bone formation and osteoclast‐mediated resorption sustains the homeostasis of bone.^[^ ^199^ ^]^ Osteoclasts, which are cells with multiple nuclei, can break down bone tissue and be tightly controlled by receptor activator of nuclear factor‐kB ligand (RANKL).^[^ ^200^ ^]^ RANKL mediates BACH1's nuclear transportation and attenuates NRF2‐mediated antioxidant enzymes, thus increasing intracellular ROS signaling and osteoclastogenesis in mice.^[^ ^201^, ^202^ ^]^ BACH1 deficiency shields against inflammatory bone loss and curtails RANKL‐mediated osteoclast formation, by inhibiting TNFα signaling and production through HO‐1 induction in macrophages adjacent to Osteoclasts.^[^ ^203^ ^]^ Additionally, HPPE, a BACH1 inhibitor, triggers BACH1 nuclear export and prompts the accumulation of NRF2 within the nucleus. This process strengthens antioxidant responses, ultimately preventing osteoclast development,^[^ ^202^ ^]^ which shows that BACH1 might be worth considering as a therapeutic target for bone destruction diseases such as osteoporosis and rheumatoid arthritis (RA).^[^ ^204^, ^205^, ^206^, ^207^ ^]^ BACH1 is crucial in bone metabolism by influencing osteoblasts and osteoclasts, highlighting the need for future development of skeletal system‐specific BACH1 inhibitors.

### 3.7 BACH1 in Odontoblasts and Periodontal Differentiation

Odontoblasts are specialized mesenchymal stem cells that form dentin, a crucial component of teeth, and play a key role in maintaining dental health.^[^ ^208^, ^209^ ^]^ BACH1 exhibits higher levels of expression in the odontoblastic layer. Under suitable environmental circumstances or particular stimulation, human dental pulp stem cells (hDPSCs) have the ability to transform into odontoblasts, thereby restoring injured dental pulp tissue.^[^ ^210^, ^211^ ^]^ Knocking out BACH1 significantly inhibits cell proliferation in hDPSCs, causing cell cycle arrest, reducing alkaline phosphatase activity, decreasing calcium deposition, and downregulating mineralization markers' expression in a manner independent of HO‐1. Thus, BACH1 promotes the proliferation and odontoblastic differentiation of hDPSCs.^[^ ^212^ ^]^ Periodontal ligament cells (PDLCs), exhibiting stem cell properties, serve as key players in the regeneration and restoration of periodontal tissue.^[^ ^213^ ^]^ Research has shown that increased BACH1 expression in PDLCs during inflammation can suppress the activity of antioxidant enzymes like HO‐1 and GCLM. Moreover, BACH1 hinders the expression of osteoblastic genes by associating with histone methyltransferase EZH2, boosting EZH2's capacity to induce H3K27me3 expression in the promoters of RUNX2 and BMP6. Therefore, silencing BACH1 alleviates inflammatory harm for PDLCs during periodontal bone regeneration.^[^ ^214^, ^215^ ^]^ The manipulation of BACH1 expression levels could unveil novel approaches for boosting the regenerative capacity of odontoblasts and periodontal tissue.

## 4 The Involvement of BACH1 in Cancer

### 4.1 BACH1 and Tumor Angiogenesis

**Figure 9:**
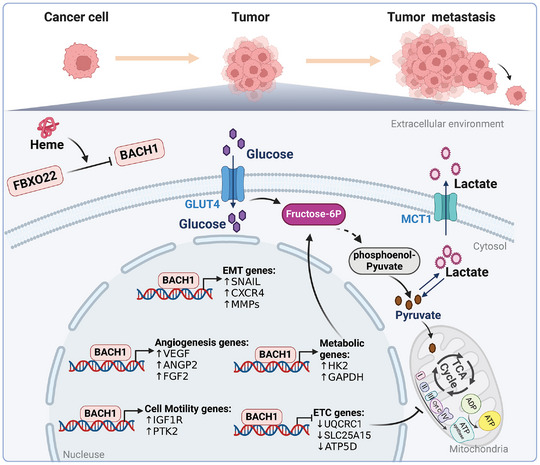
The role of BACH1 in tumor angiogenesis, metabolism, and metastasis. BACH1 plays a pivotal role in promoting tumor angiogenesis and facilitates cancer metastasis through the modulation of epithelial‐mesenchymal transition (EMT). Additionally, BACH1 enhances the expression of pro‐metastatic factors and regulates the metabolic processes of cancer cells. The illustration was generated using BioRender under a licensed subscription (Agreement number: WB27P3KHKO).

Angiogenesis in tumors, which is the formation of blood vessels to facilitate the growth of tumors, is a key factor in cancer advancement.^[^ ^216^, ^217^ ^]^ In addition to regulating angiogenesis in endothelial cells and pericytes, BACH1 also participates in tumor angiogenesis in cancer cells. The majority of studies showed that high BACH1 expression increases tumor angiogenesis (**Figure** **9**). For example, overexpression of BACH1 induces VEGFC expression significantly in human ovarian clear cell carcinoma cells. It also enhances the density of blood vessels within tumors and the diameter of lymphatic vessels surrounding tumors in ovarian and lung mouse tumor models.^[^ ^218^ ^]^ Studies in zebrafish reveal that BACH2 paralogs are necessary for developmental angiogenesis, because reduction of BACH2a impairs blood vessel formation and lymphatic sprouting during zebrafish embryonic development, which is VEGFC‐dependent.^[^ ^218^ ^]^ BACH1 also upregulates the transcription of VEGFC and facilitates tumor angiogenesis in a xenograft model of esophageal squamous cell carcinoma.^[^ ^219^ ^]^ Moreover, angiogenesis gene expression (including VEGFA, VEGFC, VEGFD, FGF2, FGFR2, EGFR, and ANGP2), as well as VEGFR2 and NRP2 protein levels are increased with high BACH1 expression in the human lung cancer cells. BACH1 mediates angiogenesis in an HIF1α‐independent way in lung cancer cells. BACH1 expression in tumor sections of lung cancer patients is relevant to the expression of angiogenic genes and proteins. Antioxidants augment tumor vascularity in vivo in a BACH1‐dependent manner, and BACH1 overexpression increases the sensitivity of tumor cells toward anti‐angiogenesis therapy.^[^ ^54^ ^]^ On the contrary, only one report showed that BACH1 decreases the protein levels of HO‐1, p‐AKT, p‐ERK, eNOS, HIF1α, and VEGF, and inhibits angiogenesis in pancreatic cancer cells.^[^ ^220^ ^]^ In ischemia‐induced angiogenesis of adult mice, BACH1 exerts its effect in inhibiting angiogenesis in endothelial cells under the condition of increased oxidative stress, which is different from the role of BACH1 in tumor cells. This discrepancy is possible because BACH1 inhibits angiogenesis genes in endothelial cells while increasing angiogenesis genes in cancer cells. This is similar to the existing research reports that BACH1 may exhibit dual roles, activating or repressing the same gene, to fine‐tune expression in response to environmental cues.^[^ ^221^ ^]^ Thus, BACH1 can exert opposite effects on angiogenesis due to differences in cell types or the extracellular microenvironments.

### 4.2 BACH1 and Tumor Metastasis

BACH1 facilitates the metastasis of several cancers, including breast cancer,^[^ ^75^, ^222^, ^223^, ^224^, ^225^, ^226^, ^227^, ^228^, ^229^ ^]^ lung cancer,^[^ ^31^, ^39^, ^230^ ^]^ pancreatic cancer,^[^ ^231^, ^232^, ^233^ ^]^ colorectal cancer,^[^ ^46^, ^234^, ^235^, ^236^, ^237^, ^238^ ^]^ ovarian cancer,^[^ ^239^, ^240^, ^241^ ^]^ glioma,^[^ ^242^, ^243^ ^]^ and renal cell carcinoma.^[^ ^244^ ^]^ The epithelial‐mesenchymal transition (EMT) serves as a determining factor in triggering cancer metastasis by suppressing epithelial gene activity and elevating mesenchymal gene activity, thereby amplifying the migratory and invasive capabilities of tumor cells.^[^ ^245^ ^]^ BACH1 suppresses the expression of forkhead box A1(FOXA1), claudin 3(CLDN3), claudin 4(CLDN4), and additional genes responsible for epithelial adhesion in pancreatic ductal adenocarcinoma cells. This inhibition disrupts the preservation of the epithelial phenotype, fosters the progression of EMT, and contributes to the malignant advancement of pancreatic cancer.^[^ ^231^, ^246^ ^]^ Our recent research findings indicate a notable rise in the expression of BACH1 within human ovarian epithelial carcinoma. The metastasis of ovarian cancer migration is promoted by BACH1 through its recruitment of high‐mobility group AT‐hook protein 2 (HMGA2) and binding to the promoters of EMT‐related genes, such as snail family transcriptional repressor 1 (SNAIL) and snail family transcriptional repressor 2 (SLUG). Furthermore, BACH1 stimulates p‐AKT and p‐p70S6K, leading to enhanced cyclin D1 expression, which facilitates the proliferation of ovarian cancer cells and transplanted tumors.^[^ ^239^ ^]^ In another study, transcriptomic data was analyzed to develop a mechanism‐based gene regulatory network, revealing that Raf kinase‐B inhibitor protein (RKIP) and BACH1 belong to two antagonistic “teams”—BACH1 driving the promotion of EMT, stem‐like, and therapy‐resistant cell states, while RKIP enabling the promotion of epithelial, less stem‐like, and therapy‐sensitive phenotypes. In numerous cancer types, low levels of RKIP and upregulated levels of BACH1 are associated with worse clinical outcomes.^[^ ^247^ ^]^ In esophageal squamous cell carcinoma, BACH1 renders CDH2, SNAI2, and VIM's upregulation by directly targeting their promoter regions, promoting the process of EMT.^[^ ^219^ ^]^ A recent study reveals that the Keto diet leads to tumor metastasis by raising ATF4 levels, which interacts with BACH1 to enhance its transcriptional activation on pro‐metastatic target promoters.^[^ ^248^ ^]^ Additionally, many other factors such as Oncogenic HOXB8,^[^ ^46^ ^]^ variable shear factor QKI,^[^ ^249^ ^]^ miR‐142‐3p,^[^ ^250^ ^]^ lncRNA AC016727.1,^[^ ^55^ ^]^ and lncRNA TRG‐AS1/miR‐4500 axis^[^ ^74^ ^]^ are capable of targeting BACH1, controlling EMT, and participating in the development of colorectal cancer, esophageal cancer, breast cancer, non‐small cell lung cancer, and liver cancer individually.

ECM remodeling and abnormal pro‐metastatic factors expression are crucial in cancer invasion and metastasis.^[^ ^251^, ^252^ ^]^ Research indicates that high levels of BACH1 in glioblastomas enhance ECM component expression and accumulation, activate the TGFBR2‐smad2/3 pathway, promote invasive protrusions, induce MMP2 expression and release, and accelerate glioma metastasis.^[^ ^78^, ^242^ ^]^ BACH1 fosters the growth and spread of hepatocellular carcinoma (HCC) by upregulating genes related to cell motility, like protein tyrosine kinase 2 (PTK2) and insulin‐like growth factor 1 receptor (IGF1R). Notably, the ligand for IGF1R, insulin‐like growth factor 2 (IGF2), increases BACH1 expression via the IGF1R‐ERK1/2‐ETS1 pathways, creating a feedback mechanism that exacerbates HCC progression and metastasis.^[^ ^50^ ^]^ Elevated BACH1 expression in colon cancer enhances cancer cell migration by upregulating the metastasis‐associated genes such as MMP‐1, MMP‐9, MMP‐13, SNAIL, CXCR4, and HMGA2.^[^ ^234^ ^]^ Upregulated BACH1 expression promotes the growth, movement, and infiltration of colorectal cancer (CRC) cells, potentially linked to higher levels of CD31, vimentin, and C‐X‐C Motif Chemokine Receptor 4 (CXCR4).^[^ ^235^ ^]^ Bioinformatic analyses in breast cancer reveal that BACH1 induces four Bone Marrow Signature (BMS) genes: MMP‐1, CXCR4, FHL1, and DUSP1.^[^ ^222^ ^]^ Specifically, BACH1 augments MMP‐1 expression by binding to its promoter, facilitating metastasis.^[^ ^75^, ^222^ ^]^ RKIP inhibits BACH1, targeting let‐7, and subsequently inhibits bone metastasis in breast cancer by downregulating BMS genes, such as MMP1, OPN, and CXCR4.**^[^** ^75^ **^]^** In brief, BACH1 facilitates cancer metastasis by modulating EMT, and ECM remodeling, as well as enhancing the pro‐metastatic factors expression. BACH1 plays a central role in cancer metastasis across various types, suggesting its wide relevance (Figure 9).

Long‐term intake of antioxidants, like N‐acetylcysteine and vitamin E, stimulates metastasis through the reduced levels of free heme and the stabilization of BACH1 in lung cancer and melanoma.^[^ ^39^, ^253^ ^]^ Elevated BACH1 levels are associated with the spread of lung cancer and reduced survival duration in individuals diagnosed with this disease.^[^ ^31^ ^]^ An additional research investigation highlighted that promoting NRF2 activity leads to increased lung cancer spread through the inhibition of BACH1 degradation. In about 30% of human lung cancer cases, mutations arise in either KEAP1 or NRF2 (encoded by Nfe2l2), leading to NRF2 stabilizing and the modulation of oxidative homeostasis.^[^ ^254^ ^]^ However, the accumulation of NRF2 in lung cancer induces HO‐1, an enzyme catalyzing heme degradation, leading to the stabilization of BACH1, which subsequently facilitates the metastasis of lung adenocarcinoma (LUAD). Antioxidants can have different effects in various conditions, with their “detrimental” effects being more noticeable in some cancers. It challenges the conventional perspective on the role of antioxidants in cancer prevention, emphasizing the necessity for judicious selection of antioxidants in clinical applications and the consideration of the network effects of molecular mechanisms. The use of ZnPPIX, an HO‐1 inhibitor, significantly reduces BACH1's expression in an FBXO22‐dependent manner to inhibit lung cancer metastasis.^[^ ^31^, ^255^ ^]^ Of even greater significance is the research demonstrating that siRNA‐mediated downregulation of BACH1 diminishes the expression of EMT and pro‐metastatic factors, thereby suppressing the metastasis of breast or colon cancer.^[^ ^234^, ^256^ ^]^ This emphasizes BACH1's importance as a key target for inhibiting cancer metastasis.^[^ ^257^ ^]^ In the future, cancer treatments might combine siRNA technology with chemotherapy or immunotherapy to boost effectiveness.

### 4.3 BACH1 and Tumor Metabolism

Tumor cells commonly exhibit heightened aerobic glycolysis and generate lactic acid as their primary metabolic alterations.^[^ ^258^, ^259^ ^]^ BACH1 and glycolytic gene expression have been shown to be positively correlated in lung cancer.^[^ ^39^ ^]^ BACH1 triggers the transcription of hexokinase 2 (HK2) and Glyceraldehyde‐3‐phosphate dehydrogenase (GADPH), leading to enhanced absorption of glucose, acceleration of glycolysis, and release of lactate, thereby driving glycolysis‐dependent metastasis in both mouse and human lung cancer cells. BACH1 controls metabolic reprogramming by upregulating the levels of HK2 and GADPH, while downregulating the expression of genes related to the mitochondrial electron transport chain. This leads to enhanced glycolysis and inhibited mitochondrial tricarboxylic acid (TCA) metabolism, both of which are characteristic features of cancer. BACH1 suppresses the mitochondrial metabolism in triple‐negative breast cancer (TNBC) cells by modulating the expression of genes related to the mitochondrial electron transport chain (ETC) in a negative manner (Figure 9). Repression of BACH1 suppresses the spread of TNBC cells to the lungs and enhances their responsiveness to metformin, indicating that specifically blocking BACH1 can alter tumor metabolism by boosting mitochondrial metabolism. Consequently, this renders tumor cells vulnerable to mitochondrial respiratory inhibitors.^[^ ^223^ ^]^ BACH1 negatively controls lactate catabolic pathways in TNBC cells as well. By inhibiting the transcriptional activation of monocarboxylate transporter 1 (MCT1) and lactate dehydrogenase B (LDHB), BACH1 impedes lactate‐mediated mitochondrial metabolism. A decline in BACH1 resulted in an upsurge in lactate consumption by TNBC cells, boosting their sensitivity to MCT1 inhibition. The combination of hemin with MCT1 inhibitors efficiently hinders the growth of TNBC.^[^ ^260^ ^]^ BACH1 not only plays a role in the glycolytic pathway but also influences tumor cell metabolic reprogramming by regulating mitochondrial metabolism and lactate metabolism. Inhibiting BACH1 could disrupt tumor cell metabolism, increasing their sensitivity to chemotherapy and other treatments.

There are various factors that can influence tumor metabolism by modulating BACH1. USP47 enhances BACH1 stability, thereby fostering the Warburg effect and the progression of non‐small cell lung cancer by boosting HK2 and GAPDH transcription.^[^ ^42^ ^]^ RCC2, a mitotic regulator, stimulates glucose metabolism in glioma by increasing HK2 expression via BACH1‐dependent transcriptional mechanisms.^[^ ^43^ ^]^ LncRNA SNHG5 induces the upregulation of BACH1 by directly targeting miR‐299, subsequently elevating the levels of HK2, PFK1, and GAPDH, and facilitating the glycolysis and proliferation of breast cancer cells.^[^ ^62^ ^]^ Given BACH1's pivotal function in tumor metabolism, the targeted manipulation of BACH1 is of paramount significance in cancer therapy. It is demonstrated that metformin inhibits papillary thyroid cancer cell growth by regulating cellular energy metabolism and increasing sensitivity through the depletion of BACH1.^[^ ^261^ ^]^ Researchers used the BACH1 inhibitor hemin and the mitochondrial function inhibitor berberine derivative (BD), to prepare nanoparticles (BH NPs). These nanoparticles were then surface‐modified with chondroitin sulfate (CS) for targeted tumor delivery, resulting in the creation of CS/BH NPs. It was found that CS/BH NPs could restrain tumor migration well and invasion in TNBC by reducing the levels of tumor cell metabolites, glycolysis, and metastasis‐related proteins.^[^ ^262^, ^263^ ^]^ Considering the challenging landscape of cancer treatment, continued exploration is essential to develop more approaches focusing on BACH1 and tumor metabolism for treating cancer.

### 4.4 BACH1 and Tumor Ferroptosis

Ferroptosis is a kind of iron‐mediated cell death triggered by lipid peroxidation.^[^ ^264^, ^265^ ^]^ Free ferrous iron (Fe^2+^) catalyzes the conversion of hydrogen peroxide to hydroxyl radicals, thereby stimulating lipid peroxidation within cellular and organelle membranes.^[^ ^266^ ^]^ The oxidation of polyunsaturated fatty acids (PUFAs) functions centrally in ferroptosis.^[^ ^266^ ^]^ Cells deploy multiple defense mechanisms to counterbalance lipid peroxidation, including the GSH‐dependent decrease of phospholipid hydroperoxides mediated by glutathione peroxidase 4 (GPX4) (GSH‐GPX4 pathway),^[^ ^267^ ^]^ restraint of labile iron via ferritin and ferroportin (ferritin‐ferroportin pathway),^[^ ^148^, ^268^ ^]^ the capture of lipophilic radicals via ubiquinol (coenzyme Q [CoQ]) and its oxidoreductase ferroptosis suppressor protein 1 (FSP1) (FSP1‐CoQ pathway),^[^ ^269^, ^270^ ^]^ along with the tetrahydrobiopterin (BH4) system.^[^ ^271^ ^]^

BACH1 inhibits the expression of key genes that regulate ferroptosis pathways, including the GSH‐GPX4 pathway, intracellular labile iron metabolism, and the FSP1‐CoQ pathway,^[^ ^272^, ^273^, ^274^, ^275^, ^276^, ^277^ ^]^ thereby promoting ferroptosis. (a) SLC7A11 inhibition reduces cysteine availability for GSH biosynthesis, leading to lower GPX4 activity. BACH1 suppresses SLC7A11 expression and also inhibits the subunit genes of the key enzyme in the GSH synthesis pathway: glutamate‐cysteine ligase modifier subunit (GCLM) and glutamate‐cysteine ligase catalytic subunit (GCLC).^[^ ^148^ ^]^ (b) Ferritin proteins encoded by FTH1 (ferritin heavy chain 1) and FTL (ferritin light chain) neutralize intracellular labile Fe^2+^ by storing it within their spherical structure. Ferroportin, a SLC40A1 (solute carrier family 40 member 1)‐encoded transporter protein regulates cellular iron homeostasis, which helps maintain optimal levels of iron within the cell. The suppression of FTL, FTH1, and SLC40A1 by BACH1 leads to an upsurge in the intracellular labile iron content, ultimately supporting the progression of ferroptosis.^[^ ^148^, ^278^, ^279^, ^280^, ^281^ ^]^ It is intriguing that BACH1 decreases HO‐1 activation and hinders Fe^2+^ accumulation, providing a protective shield against ferroptosis. Conversely, when HO‐1 is elevated due to reduced BACH1 levels, enough ferritin sequesters Fe^2+^ produced from heme breakdown to protect cells from ferroptosis. Therefore, BACH1‐induced HO‐1 modulation may have a role in inhibiting or upregulating ferroptosis, relying on the balance of HO‐1 and ferritin.^[^ ^282^, ^283^ ^]^ (c) FSP1 downregulates ubiquinone (CoQ), thus inhibiting lipid peroxidation in cellular membranes. Whether BACH1 induce ferroptosis through interacting with NRF2 and mediating the FSP1‐CoQ pathway and tetrahydrobiopterin (BH4) system is worth studying.^[^ ^272^ ^]^ Additionally, BACH1 facilitates ferroptosis via transcriptionally regulating genes in lipid metabolism as well. ACSL4 (acyl‐CoA synthetase long‐chain family member 4), an enzyme that aids in generating PUFA‐PLs, is controlled by the coordination of cells through cadherin‐mediated contacts. BACH1 represses E‐cadherin expression and activates ACSL4 expression by indirect interference with the Neurofibromin 2 (NF2)‐Hippo pathway.^[^ ^272^ ^]^

Tumor ferroptosis is intricately linked to metastasis.^[^ ^284^, ^285^ ^]^ BACH1 suppresses the transcription of Stearoyl‐CoA desaturase‐1 (SCD1) which shields tumor cells from ferroptosis,^[^ ^286^ ^]^ leading to a decrease in oleic acid production and subsequently lowers resistance to ferroptosis.^[^ ^287^ ^]^ In Esophageal squamous cell carcinoma (ESCC), ferroptosis mediated by BACH1 actuates lymphatic metastasis through the BACH1‐SCD1‐OA axis.^[^ ^287^ ^]^ BACH1 promotes glioma invasion primarily by modulating the extracellular matrix and increasing its sensitivity to ferroptosis conversely.^[^ ^242^ ^]^ TBK1 promotes the expression of BACH1 in pancreatic cancer cells, leading to elevated iron levels and decreased E‐Cadherin expression, which in turn enhances the metastasis of cancer cells.^[^ ^288^ ^]^ The p53^R175H^ mutant protein, a hotspot variant of p53, selectively binds to BACH1, counteracting the BACH1‐induced decrease in SLC7A11 expression, consequently suppressing ferroptosis and facilitating tumor proliferation.^[^ ^15^ ^]^ Bile acids block BACH1 by activating the farnesoid X receptor, reduce ferroptosis sensitivity, and promote gastric cancer progression.^[^ ^289^ ^]^ Sanguinarine chloride, a benzophenanthrine alkaloid derived from certain plant roots, triggers ferroptosis in prostate cancer through the ROS/BACH1/HMOX1 pathway, inhibiting prostate cancer growth effectively.^[^ ^290^ ^]^ Ferroptosis can be more easily induced in tumor cells due to their increased need for iron in contrast to other tissue cells. By activating ferroptosis through diverse mechanisms, BACH1 might offer a potential treatment strategy for cancers. Tumors with increased BACH1 expression may show a greater sensitive to ferroptosis‐inducing drugs compared to those with decreased levels, and further experimentation is required for validation.

### 4.5 BACH1 and Tumor Microenvironment

The tumor microenvironment (TME) includes cancer cells, non‐malignant cell types, and altered extracellular matrix.^[^ ^291^ ^]^ The immune response in TME is necessary for cancer growth and advancement.^[^ ^292^ ^]^ In TME, tumor cells can manipulate the activity of immune cells, suppress the attack of immune cells on cancer cells, and evade immune surveillance by the body.^[^ ^293^, ^294^ ^]^ BACH1 has been increasingly recognized for its involvement in tumor immunity.^[^ ^295^, ^296^ ^]^ BACH1 is strongly expressed in many cancers, often correlating with bad prognosis. In TNBC, upregulated BACH1 expression is strongly linked to circulating tumor cells (CTCs) and monocyte‐derived dendritic cells (Mo‐MDCs), contributing to a negative outcome.^[^ ^297^ ^]^ The protein APOBEC3A (A3A), originating from an essential enzyme in the innate immune system, causes mutations in breast cancers by altering cytidines in single‐stranded DNA to uridine.^[^ ^298^ ^]^ BACH1 elevates basal A3A expression leading to breast cancer mutagenesis, further promoting cancer progression and metastasis.^[^ ^299^ ^]^ Research indicate a positive correlation between BACH1 expression and the amount of tumor‐infiltrating lymphocytes (TILs) in various types of cancer.^[^ ^300^ ^]^ BACH1‐related gene expression for functional enrichment analysis is involved in processes such as ubiquitin‐driven proteolysis, activation of T cell receptors, modulation of PD‐1/PD‐L1 expression, differentiation of Th17 cells, and endocytic mechanisms.^[^ ^300^ ^]^ The TISCH database reveals that BACH1 expresses significantly in CD4^+^ T cells, CD8^+^ T cells, B cells, monocyte‐macrophages, and malignant cells in a majority of tumors.^[^ ^301^ ^]^ BACH1 inhibits the effective response of the immune system, helping tumors evade immune surveillance, and thereby promoting tumor growth and metastasis.

Macrophages polarize to the M2 phenotype, increase immune tolerance, and induce gastric tumorigenesis.^[^ ^302^, ^303^ ^]^ LINC00665 interacts with BACH1 to activate Wnt1 and triggers the M2 polarization, facilitating macrophage‐dependent GC progression.^[^ ^179^ ^]^ BACH1 is active in the TGM2^+^ macrophage subtype, which promotes macrophage anti‐inflammatory responses in pancreatic ductal adenocarcinoma (PDAC).^[^ ^304^ ^]^ BACH1 governs most effector and memory HBV‐specific T cells, shaping T‐cell immune responses in HBV‐related hepatic cell carcinoma (HCC) as well.^[^ ^305^ ^]^ BACH1 contributes to the development of an immunosuppressive tumor microenvironment in glioblastoma, associated with enhanced immune responses by upregulating the expression of immune checkpoints (ICs), chemokines produced by tumor‐associated macrophages (TAMs), and M2 TAMs markers.^[^ ^306^ ^]^ PDZ‐LIM domain‐containing protein 2 (PDLIM2), is a pivotal checkpoint for alveolar macrophages (AMs), playing a crucial role in lung tumor suppression. BACH1 inhibits the expression of PDLIM2, driving alveolar macrophages (AM) protumorigenic polarization/activation and differentiating from circulation‐attracted monocytes to impair cytotoxic T lymphocytes and facilitate lung cancer.^[^ ^307^ ^]^ LncRNA BACH1‐IT2's high presence in bladder cancer correlates with reduced miR‐4786, significantly impacting the high levels of Siglec‐15 (sialic acid binding Ig like lectin 15), a previously identified tumor immunosuppressive gene that speeds up immune evasion in bladder cancer.^[^ ^308^ ^]^ Collectively, BACH1 hinders the innate and acquired immune systems in various cancers, facilitating the M2 phenotype switch of macrophages and the development of an immunosuppressive tumor microenvironment, ultimately fostering cancer progression. Consequently, focusing on controlling BACH1 regulation in the tumor microenvironment is seen as a potential method for combating tumors. Research has shown that the loss of BACH1 enhances the anti‐tumor effect on mantle cell lymphoma cells by stimulating intrinsic antitumor immune responses and inhibiting cell cycle arrest.^[^ ^309^ ^]^ Another study suggests a thermosensitive gel‐nano system for EC treatment through small interfering RNA targeting BACH1 and p53 activator PRIMA‐1, restoring anti‐tumor immune responses, has potential for clinical application.^[^ ^310^ ^]^ Further research is needed on the regulatory mechanism of BACH1 in the TME as an anti‐tumor therapy.

## 5 The Function of BACH1 in Gastrointestinal Disorders

### 5.1 BACH1 and Viral Hepatitis, NAFLD, or Hepatic Injury

Hepatitis C virus (HCV) infection is a common viral infection that can lead to liver diseases.^[^ ^311^, ^312^ ^]^ Several key observations indicate that decreased BACH1 and increased HO‐1 levels were crucial in mitigating the cytotoxic effects of HCV proteins.^[^ ^313^, ^314^, ^315^ ^]^ MiR‐let‐7c and miR‐196 inhibit BACH1 expression to impede viral protease activity and enhance antiviral interferon response.^[^ ^314^, ^315^ ^]^ Legalon‐SIL (LS) and statins inhibited HCV replication via BACH1/HO‐1 signaling as well.^[^ ^316^, ^317^ ^]^ However, another study has validated that the expression of HO‐1 in HCV patients or those co‐infected with HCV and HIV is positively associated with BACH1 and miR‐122.^[^ ^318^ ^]^ This seems to contradict the notion of BACH1 inhibiting HO‐1 expression, so further research is needed to expound the possible mechanisms. BACH1 also plays a significant role in HBV‐induced hepatitis.^[^ ^305^, ^319^ ^]^ The control of HBV infection relies heavily on CD8^+^ T cells.^[^ ^320^ ^]^ The high activity of BACH1 is observed in HBV‐specific effector and memory T cells.^[^ ^305^ ^]^ Inhibition of CircBACH1, a circular RNA derived from the BACH1 gene, showed suppressive impacts on HBV replication and hepatocellular carcinoma progression by influencing the miR‐200a‐3p/MAP3K2 axis.^[^ ^319^, ^321^ ^]^ Thus, even with conflicting findings, BACH1 is crucial in HCV and HBV infections and remains promising for antiviral treatment.

Non‐alcoholic fatty liver disease (NAFLD), associated with metabolic syndrome, poses a serious health threat due to obesity, type 2 diabetes, high cholesterol, and hypertriglyceridemia.^[^ ^322^ ^]^ Increased BACH1 is found in the hepatocytes of individuals with obesity and patients with NAFLD.^[^ ^323^ ^]^ Global deletion of BACH1 provided liver protection in mice on a methionine‐choline deficient diet.^[^ ^87^ ^]^ Instead, the deletion of hepatocyte‐specific Sirtuin 6 (Sirt6) led to an increased risk of non‐alcoholic steatohepatitis due to elevated BACH1 expression.^[^ ^324^ ^]^ Further research should clarify BACH1's regulatory mechanisms and interactions with metabolism‐related molecules to assess its potential in NAFLD therapy.

BACH1 is implicated in liver damage caused by a variety of injurious stimuli.^[^ ^325^ ^]^ The single nucleotide polymorphisms (SNPs) of BACH1 show a significant correlation with anti‐tuberculosis drug‐induced hepatotoxicity (ATDH), involving the nucleotide positions rs372883, rs1153285, and rs2070401.^[^ ^326^, ^327^, ^328^ ^]^ Besides, XPO1 SNPs are also associated with the risk of ATDH, possibly due to its interaction with BACH1 and its influence on its antioxidative activity.^[^ ^329^ ^]^ In the aryl hydrocarbon receptor (AhR)‐mediated hepatotoxicity mouse model, BACH1 expression is upregulated and associated with AhR‐mediated hepatotoxicity.^[^ ^330^ ^]^ BACH1 deficiency protects against LPS and Sepsis‐induced liver injury.^[^ ^331^, ^332^ ^]^ Reduced nuclear BACH1 levels exhibit protective effects against acute CCl4, Aflatoxin B1 (AFB1), inorganic arsenic‐induced liver damage.^[^ ^333^, ^334^, ^335^, ^336^ ^]^ By reprogramming energy metabolism and exacerbating oxidative stress, BACH1 impairs the regeneration of the liver with repeated ischemia/reperfusion after hepatectomy.^[^ ^337^ ^]^ Many studies indicate that inhibiting BACH1 has a therapeutic effect on liver injury. Diminishing BACH1 level helps vitamin D and omega‐3 fatty acids to offer protection against APAP‐induced liver injury.^[^ ^338^, ^339^ ^]^ San Wei Gan Jiang San (SWGJS) alleviates liver injury via NRF2/BACH1 as well.^[^ ^340^ ^]^ Besides, miR‐27a‐5p suppresses apoptosis by targeting and inhibiting BACH1, thus alleviating liver injury caused by ischemia‐reperfusion.^[^ ^60^ ^]^ Therefore, BACH1 is an underlying therapeutic target in various injurious stimuli‐induced hepatotoxicity.

### 5.2 BACH1 and Intestinal Diseases

Inflammatory Bowel Disease (IBD) is a chronic intestinal disorder, predominantly including ulcerative colitis (UC) and Crohn's disease (CD).^[^ ^341^ ^–^ ^343^ ^]^ These entail enduring intestine inflammation and may result in symptoms such as diarrhea, abdominal pain, anemia, and weight loss.^[^ ^344^ ^]^ A study in bioinformatics revealed that 9 crucial genes (CXCL9, TIMP1, PTGS2, ICAM1, CXCL1, MMP9, IL1B, CXCL8, and IL6) are linked to the Mayo score in ulcerative colitis, with BACH1 serving as a vital transcription factor regulating their expression.^[^ ^345^ ^]^ BACH1 deficiency alleviates dextran sodium sulfate (DSS)‐induced colitis by upregulating HO‐1 expression.^[^ ^346^ ^]^ The miR‐23a‐27a‐24‐2 cluster binds to the 3′UTR and decreases BACH1 expression, thus relieving BACH1‐induced repression of HO‐1 and exerting a therapeutic effect against DSS‐induced colitis.^[^ ^347^ ^]^ BACH1 deficiency relieves 2,4,6‐trinitrobenzene sulfonic acid (TNBS)‐mediated colitis through upregulation of HO‐1 and M2 polarization anti‐inflammatory response in macrophages.^[^ ^85^ ^]^ However, another study showed that BACH1 deficiency in mouse macrophages alters the energy metabolism of mitochondrion (upregulated glycolysis and downregulated oxidative phosphorylation), enhancing mitochondrial membrane potential and the production of mitochondrial reactive oxygen species (mtROS), while also reducing mitophagy levels.^[^ ^180^ ^]^ When exposed to LPS, macrophages lacking BACH1 demonstrate increased activation of NLRP3 inflammasomes, resulting in elevated release of pro‐inflammatory factors like IL‐1β, and IL‐18.^[^ ^180^ ^]^ This seems to be contradictory to the previous finding that BACH1 deficiency results in macrophages exhibiting an M2 anti‐inflammatory phenotype attributed to elevated HO‐1. Due to HO‐1′s critical role in macrophage iron metabolism,^[^ ^348^ ^]^ BACH1 reduction may affect the inflammatory and metabolic characteristics of macrophages, contingent upon iron pathways.^[^ ^349^ ^]^ The unusual phenotype of LPS‐induced macrophages lacking BACH1, characterized by both pro‐inflammatory (inflammasome activation) and anti‐inflammatory (HO‐1 and M2 markers) features, may reflect an intermediate state of macrophage activation. Thereby, BACH1 seems to play a dual role in intestinal inflammation.

Inhibition of BACH1 confers a protective role in different types of intestinal damage. The global use of non‐steroidal anti‐inflammatory drugs (NSAIDs) is associated with the side effect as gastrointestinal damage, also termed NSAID‐associated enteropathy.^[^ ^350^ ^]^ Studies indicate that BACH1 deficiency inhibited apoptosis in domethacin‐induced intestinal injury.^[^ ^351^, ^352^ ^]^ Besides, BACH1 deletion alleviates intestinal ischemia‐reperfusion injury by suppressing NF‐κB signaling and adhesion molecules to inhibit inflammation.^[^ ^353^ ^]^ The role of BACH1 in gastrointestinal functional disorders needs further study.

## 6 BACH1 and Neurodegenerative Disease

### 6.1 BACH1 and Down Syndrome with Alzheimer's Disease

Neurodegenerative diseases are a group of diseases that seriously threaten human health, and their main characteristics are the progressive loss and functional impairment of neurons.^[^ ^354^, ^355^ ^]^ Down syndrome (DS) or trisomy 21 is a common genetic disorder that impacts an individual's physical growth and cognitive advancement. DS patients have a higher risk of developing dementia similar to Alzheimer's disease (AD), with amyloid plaques and neurofibrillary tangles.^[^ ^356^ ^]^ BACH1, encoded on Hsa21, functions critically in regulating the antioxidant response in DS.^[^ ^357^, ^358^, ^359^ ^]^ BACH1 was significantly overexpressed in fetal and human DS subjects, before or after AD development.^[^ ^359^, ^360^ ^]^ DS individuals are characterized by enhanced oxidative/nitrosative stress (OS/NS) in early stage due to the trisomy of BACH1 inhibiting antioxidant genes like HO‐1.^[^ ^361^, ^362^ ^]^ HO‐1 deficiency leads to a decreased activity of biliverdin reductase‐A(BVR‐A) by increasing its protein nitration, which convert biliverdin, derived from the breakdown of heme by HO‐1, into bilirubin possessing strong antioxidant activity. Inhibition of HO‐1/BVR‐A pathway leads to the inability of its neuroprotective function, which is conducive to AD pathology progression in DS individuals.^[^ ^359^, ^360^ ^]^ Treatment with Caffeic Acid Phenethyl Ester (CAPE) and its synthetic analog VP961, known for their ability to modulate NRF2 activity and facilitate NRF2 nuclear translocation, leading to enhanced antioxidant response in Ts2Cje mice (a Down syndrome mouse model) and human Down syndrome lymphoblastoid cell lines (LCLs).^[^ ^362^ ^]^ Hence, targeting BACH1 and oxidative stress pathways could provide therapeutic strategies for the patients.

Besides, BACH1 also binds to the promoter of the microtubule‐associated protein tau (also known as MAPT) gene and represses its expression,^[^ ^363^ ^]^ which is associated with the progression of AD.^[^ ^364^, ^365^ ^]^ MAPT facilitates microtubule assembly and stability, establishing and maintaining neuronal polarity. Excessive MAPT phosphorylation will weaken its ability to bind to microtubules, leading to AD. Previous evidence shows that ubiquitin‐specific peptidase 9 (USP9) facilitates MAPT phosphorylation via the deubiquitination of the kinase MARK4 and synuclein alpha (SNCA).^[^ ^366^ ^]^ Recently, A transcriptome‐wide analysis discovers genes in public post‐mortem brain tissue of AD patients, identifying that USP9 is a candidate gene with gender‐specific expression and changes.^[^ ^367^ ^]^ A gender difference exists in the occurrence and phenotypic expressions of brain disorders such as AD, and women exhibit a higher AD prevalence.^[^ ^368^ ^]^ USP9 promotes the expression of MAPT by deubiquitinating SMAD4 and inhibiting the expression of BACH1, which, in turn, suppresses MAPT transcription.^[^ ^363^, ^369^ ^]^ BACH1 expression contributes to the molecular gender differences observed in tauopathies and AD, providing new avenues for intervention targeting MAPT.

### 6.2 BACH1 and Parkinson's Disease

Parkinson's Disease (PD) is a prevalent disorder characterized by the degeneration of the nervous system.^[^ ^370^ ^]^ Its clinical symptoms include resting tremor, postural instability, and bradykinesia.^[^ ^371^ ^]^ The primary pathological lesion is the loss of dopaminergic neurons in the substantia nigra pars compacta of the midbrain, leading to reduced dopamine levels in the nigrostriatal pathway.^[^ ^372^, ^373^ ^]^ The remaining dopaminergic neurons contain Lewy bodies, mainly composed of misfolded α‐synuclein.^[^ ^374^ ^]^ Oxidative stress, mitochondrial dysfunction, and inflammatory responses are considered important factors for Parkinson's disease pathogenesis. A bioinformatics analysis, focusing on differentially expressed genes (DEGs) interacting with established PD‐related genes (PDAGs) to prioritize candidate genes, identified BACH1 as a significant candidate gene.^[^ ^375^ ^]^ The protein level of BACH1 is promoted in the substantia nigra pars compacta (SNPC) of human postmortem PD. Parkinsonian neurotoxin MPTP and its toxic metabolite MPP^+^ lead to neurodegeneration through mediating oxidative stress, neuroinflammation, and mitochondrial dysfunction. BACH1 expression is also markedly upregulated in the ventral midbrains (VBMs, the brain region containing SNPC) of mice treated with MPTP. In both acute and sub‐acute models, the absence of BACH1 in mice results in a reduction of dopaminergic neuronal cell death, which is facilitated by MPTP. Ablation of BACH1 reduces MPTP‐induced oxidative stress and neuroinflammation. Pathway analysis shows that deletion of BACH1 activates ARE‐mediated neuroprotective routes that are vital for the regulation of oxygen sensing and prevention of neuronal death. Interestingly, many up‐regulated genes in BACH1 knockout mice lack an ARE motif and are classified as non‐ARE genes. Most non‐ARE genes controlled by BACH1 are transcription factors or proteins that affect the survival of neuronal cells, which may potentially benefit Parkinson's patients.^[^ ^376^ ^]^ This indicates that BACH1 may directly regulate other genes that do not participate in the oxidative stress responses, possibly in various pathways involved in neuroprotection.

### 6.3 BACH1 and Multiple Sclerosis

Multiple sclerosis (MS) is an inflammatory disease characterized by an immune‐mediated attack on oligodendrocytes (OLG) and myelin. This results in demyelination, scarring, and a wide range of signs and symptoms.^[^ ^377^, ^378^ ^]^ It is the most widespread demyelinating disease and the main reason for neurological disability among young adults.^[^ ^379^ ^]^ Microarray gene expression profiling combined with bioinformatics analysis identified BACH1 as a candidate gene for regulating MS.^[^ ^380^, ^381^ ^]^ Deficiency of BACH1 in mice with experimental autoimmune encephalomyelitis (EAE), which is a mouse model of multiple sclerosis (MS) in humans, results in less inflammation in the central nervous system (CNS) through the HO‐1‐dependent mechanism.^[^ ^382^ ^]^ Nevertheless, an epigenomic study of immune genes implicates oligodendrocytes in multiple sclerosis susceptibility, explaining the new role of BACH1 in MS. Without the presence of a disease, the immune genes in oligodendroglia (OLG) of mice and humans exhibit a primed chromatin state, which is in line with the shifts to immune‐competent states associated with MS. In the adult CNS, oligodendrocyte precursor cells (OPCs) are recruited to MS lesions, transition to an immune‐like state, and are considered to play a role in remyelination during disease alleviation. BACH1 and STAT1 were identified as transcription factors in IFN‐γ‐mediated immune gene regulation in OPCs. In primary OPCs, knockdown of BACH1 using siRNA resulted in upregulation of MHC‐I genes, such as H2‐Q4 and H2‐Q7, and MHC‐II pathway gene Cd74 under the stimulation of IFN‐γ, indicating that some immune genes are negatively regulated by BACH1 in OPCs in response to IFN‐γ‐mediated induction.^[^ ^383^ ^]^ STAT1 is a primary transducer in the IFN‐γ signaling pathway in immune cells.^[^ ^384^, ^385^ ^]^ Unlike the BACH1 Knockdown, STAT1 is responsible for upregulating immune genes mediated by IFN‐γ in OPCs.^[^ ^383^ ^]^ Therefore, other than immune cells, targeting the immune regulation of oligodendrocytes constitutes a new target for immunological‐based therapies of MS.

### 6.4 BACH1 and Cerebral Stroke or Injurious Stimulus

The degree of neuronal necrosis after cerebral ischemia contributes significantly to the high mortality and disability rates associated with ischemic stroke.^[^ ^386^ ^]^ Neurons in the ischemic core eventually die as a result of sustained ischemia or ischemic‐reperfusion injury.^[^ ^387^ ^]^ In contrast, the area surrounding the ischemic core, known as the penumbra, bears no obvious neuronal death, which can be salvaged upon restored blood flow.^[^ ^388^ ^]^ However, the penumbra may turn into an infarct in some cases, and this process is influenced by immune inflammation mediated via microglia.^[^ ^389^, ^390^ ^]^ Recently, a study used single‐cell RNA sequencing (scRNA‐seq) and spatial transcriptomics (ST) to analyze middle cerebral artery occlusion (MCAO) mice at three‐time points to identify the microglial subpopulations related to stroke and their spatial distribution. Two cell populations were defined as ischemic core‐associated (ICAM) and ischemic penumbra‐associated (IPAM) microglia. Among them, ICAM can induce excessive neuroinflammatory responses and aggravate brain damage, while IPAM may have a neuroprotective effect. BACH1 is a critical transcription factor facilitating the ICAM production. Knocking down BACH1 alleviates the inflammatory phenotype of microglia.^[^ ^391^ ^]^ Another bioinformatics analysis on immune infiltration and the construction of a competing endogenous RNA (ceRNA) network related to programmed cell death identified the apoptosis‐associated gene BACH1 as a potential target for ischemic stroke treatment.^[^ ^392^ ^]^ BACH1 promotes ferroptosis via actuating KDM4C‐induced COX2 demethylation under cerebral ischemia‐reperfusion injury.^[^ ^393^ ^]^ Reduced BACH1 expression protects against middle cerebral artery occlusion (MCAO) and cerebral ischemia/reperfusion injury.^[^ ^393^, ^394^, ^395^ ^]^ By suppressing BACH1, miR‐532‐5p, miR‐98‐5p, and N‐hydroxy‐N'‐(4‐butyl‐2‐methylphenyl)‐formamidine offer protection from neurological deficits following ischemic stroke.^[^ ^396^, ^397^, ^398^ ^]^ Meanwhile, Circular RNA (circZfp609) and LncRNA X‐inactive specific transcript (XIST) facilitate the development of cerebral infarction through the regulation of the miR‐145a‐5p/BACH1 pathway and miR‐98/BACH1 Axis individually.^[^ ^399^, ^400^ ^]^ Overall, BACH1 plays a complex role in cerebral ischemic injury. Future studies may elucidate its specific mechanisms and provide new therapeutic strategies for clinical applications.

BACH1 serves a crucial role in the presence of harmful nerve stimulation as well. A widely used fungicide, Maneb (MB), belongs to the dithiocarbamate pesticides family.^[^ ^401^ ^]^ One environmental trigger of the multifactorial neurodegenerative disease has been identified as exposure to MB.^[^ ^402^ ^]^ Research indicates that mild overexpression of α‐synuclein inhibits MB‐mediated neurotoxicity by reducing nuclear BACH1 expression and modulating NRF2 and FOXO3a transcription factors, and probably interferes with ferroptosis‐related mechanisms to prevent cell death.^[^ ^403^ ^]^ Exposure to lead (Pb), a known neurotoxic substance that causes developmental neurotoxicity, can result in impairments of neurogenesis.^[^ ^404^ ^]^ The deletion of BACH1 reversed the Pb‐mediated transcriptional and expressive decrease of genes associated with oxidative phosphorylation (OXPHOS) and eliminated the growth suppression of axon activation and synapse formation induced by Pb.^[^ ^405^ ^]^ BACH1 deficiency leads to high expression of HO‐1 and a significant increase in cytoprotection against spinal cord injury as well.^[^ ^91^ ^]^ Exosomes differentiated from mesenchymal stem cells alleviate neuronal injury caused by oxygen‐glucose deprivation/ reoxygenation by transferring miR‐194 and downregulating BACH1.^[^ ^406^ ^]^ Neural development also involves BACH1. During mouse brain development, BACH1 modifies microglial metabolism and influences astrogenesis, while microglia‐derived LRRC15 collaborates with CD248 as part of the JAK/STAT pathway and affect astrogenesis, and microglial BACH1‐deficient mice display neuronal differentiation abnormalities and anxiety‐like behaviors.^[^ ^407^ ^]^ These findings offer a solid foundation for further validating the therapeutic potential of BACH1 in neuro‐related diseases.

## 7 BACH1 and Diabetes

Diabetes is a chronic metabolic disorder known by a persistent state of high glucose in the blood, influenced by various genetic and environmental factors that impact the pancreatic β‐cells’ function and insulin signaling pathways.^[^ ^408^, ^409^ ^]^ BACH1 deficiency inhibits alloxan‐induced reduction in pancreatic insulin content and the resulting glucose elevation by reducing the apoptosis of pancreatic β‐cells under oxidative stress.^[^ ^94^ ^]^ Insulin resistance implies reduced insulin reactivity, and ineffective glucose uptake/utilization, linked to conditions like type 2 diabetes, non‐alcoholic fatty liver disease (NAFLD), cardiovascular disease, etc. Long‐term exposure to elevated free fatty acids, like palmitate, results in β‐cell failure and contributes to insulin resistance. BACH1 modulates the metabolic and oxidative stress reactions to palmitate, which contributes to the dysfunction of human pancreatic β‐cells in the presence of palmitate.^[^ ^410^ ^]^ Our research also shows that BACH1 negatively regulates insulin signaling. BACH1 interacts directly with the insulin receptor β (IR‐β) along with the protein‐tyrosine phosphatase 1B (PTP1B), which dephosphorylating the insulin receptor‐β (IR‐β) to repress IR‐β‐induced insulin signaling. BACH1 absence decreases the interaction of PTP1B with IR‐β under insulin induction, leading to consequent enhancement of insulin signaling in hepatocytes. As a result, hepatocyte‐specific BACH1 deficiency elevates insulin signaling and alleviates abnormal glucose homeostasis regulation in high‐fat diet (HFD)‐caused hepatic insulin resistance, and finally prevents individuals from HFD‐induced steatosis.^[^ ^323^ ^]^ Instead, deletion of Progesterone receptor membrane component 2 (PGMRC2) in brown fat, an intracellular haem chaperone, decreases labile haem in the nucleus and improves the stability of BACH1, which is the haem‐responsive transcriptional suppressor, resulting in higher fasting glycemia, reduced glucose tolerance and insulin sensitivity, hyperlipidemia, and exacerbated liver steatosis in mice with HFD.^[^ ^195^ ^]^ These studies revealed the adverse role of BACH1 in insulin resistance and diabetes.

The occurrence and progression of cardiovascular complications in diabetic patients are associated with endothelial‐to‐mesenchymal transition (EndMT).^[^ ^411^ ^]^ Hyperglycemia/high glucose increases BACH1 expression in the aorta of diabetic rats, and BACH1 accelerates hyperglycemia‐induced EndMT via facilitating AKT3 transcription, promoting diabetic cardiovascular complications.^[^ ^412^ ^]^ Consistently, another research shows that SETD8 interacts with ELK1 to inhibit the transcription of BACH1, negatively regulating EndMT in diabetic nephropathy (DN), one of the most common complications among diabetic patients, which causes kidney failure.^[^ ^413^ ^]^ A bioinformatics analysis of patients with DN identified a negative connection between BACH1 level and glomerular filtration rate (FGR), indicating that BACH1 may have a nephrotoxic function in DN.^[^ ^414^ ^]^ The examination of chromatin accessibility and the architectural profiling of human kidneys identify BACH1 as a key transcription factor in damaged proximal tubule (PT) cells, with its target genes having a high correlation with fibrosis and inflammation.^[^ ^415^ ^]^ Circ_012 3996 induces BACH1 expression in mice mesangial cells by sponging miR‐149‐5p to promote DN.^[^ ^416^ ^]^ These studies show that BACH1 is closely linked to cardiovascular complications and diabetic nephropathy in diabetes. The development of BACH1 inhibitors may present a new therapeutic option for insulin resistance, diabetes, and related complications.

## 8 BACH1 and Respiratory Diseases

BACH1 facilitates the progression and metastasis of lung cancer and is additionally implicated in various other respiratory diseases. BACH1 is increased in cystic fibrosis and chronic obstructive pulmonary disease (COPD) patients and is linked to respiratory impairment.^[^ ^417^, ^418^ ^]^ BACH1 additionally increases mycobacterium tuberculosis susceptibility and tissue necrosis.^[^ ^419^ ^]^ Idiopathic Pulmonary fibrosis (IPF) is a chronic and advancing disease featuring diffuse inflammation, interstitial fibrosis, and the distortion of the lung framework, resulting in extensive impairment and dysfunction of the lungs.^[^ ^420^ ^]^ Oxidative stress is essential for inflammation and fibrosis.^[^ ^421^ ^]^ For the important function of BACH1/NRF2 signaling in oxidative stress, BACH1 siRNA impedes bleomycin (BLM)‐mediated pulmonary fibrosis in mice through regulating oxidative stress.^[^ ^90^ ^]^ Pirfenidone (PFD), a sanctioned medication for treating IPF, significantly improves the life quality and pulmonary function of IPF patients. PFD's therapeutic effects on IPF involve the antioxidant mechanism of suppressing BACH1 and enhancing NRF2.^[^ ^422^ ^]^ BACH1 is also involved in pulmonary fibrosis through other mechanisms. Studies have shown that BACH1 suppression relieves fibrosis and inflammation by blocking the ERK signaling.^[^ ^423^ ^]^ BACH1 transcriptionally enhances FOSL2, thereby inducing the M2 macrophage phenotype through activating the TGFβ/SMAD signaling to facilitate the transformation of lung fibroblasts into myofibroblasts, and BACH1 deficiency alleviates BLM‐mediated pulmonary fibrosis.^[^ ^424^ ^]^ These studies suggest that BACH1 seems to be detrimental in lung fibroblast cells, and inhibition of BACH1 can alleviate pulmonary fibrosis. However, a study revealed increased nuclear level of BACH1 in pathologic mesenchymal progenitor cells (MPCs), which causing sustained interstitial lungs fibrosis in IPF patients, by Quantitative mass spectrometry studies combined with interactomic analysis. While silencing of BACH1 enhances the survival of IPF MPCs, potentially accelerating the progression of IPF.^[^ ^425^ ^]^ This implies that even for the same disease, the function of BACH1 in different tissue cells might be distinct, and in the future treatment strategy of the disease, targeted intervention of BACH1 expression levels in specific cells is essential (**Table** **2**).

**Table 2:** Functions of BACH1 in various diseases.

Disease		BACH1 alteration	Target genes	Functions	Refs.
Cardiovascular Disease	Ischemic angiogenesis	Upregulated	Antioxidant genes (HO‐1, PGC1α) Angiogenesis genes (VEGF, IL‐8, ANGP‐1, FGF2)	Increased ROS production and apoptosis in ECs Inhibiting angiogenesis by decreasing angiogenesis related genes	[11, 59, 97, 115, 116, 117, 118, 119]
	Atherosclerosis	Upregulated	Inflammation related genes (ICAM1, VCAM1) Antioxidant genes (HO‐1)	BACH1 deficiency reduces atherosclerotic lesion formation through the anti‐inflammatory effect by inhibiting YAP activation in ECs Through HO‐1‐dependent anti‐oxidative effects.	[14, 125, 126, 127]
	Restenosis	Upregulated	VSMC marker gene (ACTA2, CNN1) NAD(P)H genes (NOX2)	BACH1 deficiency reduces intimal hyperplasia through maintaining VSMC phenotype and promoting endothelial regeneration.	[11, 13, 132, 133, 135, 136]
	Myocardial hypertrophy	Upregulated	AT1R Ca^2+^/CaMKII pathway Antioxidant genes (HO‐1)	Loss of BACH1 mitigates cardiac hypertrophy caused by Ang II and pressure overload, reduces cardiac fibrosis, and maintains cardiac function	[49, 142]
	I/R injury	Upregulated	Antioxidant genes (HO‐1, NQO1) STAT3 pathway p38/MAPK signaling	BACH1 deficiency reducing damage caused by oxidative stress during I/R cardioprotective ability of BACH1 disruption is also associated with STAT3 activation and inhibition of p38/MAPK signaling and apoptosis in myocardial tissues	[61, 88, 145, 146]
	Myocardial infarction	Upregulated	LncRNA AZIN2‐sv ferroptosis genes (FTN, FPN, and HO‐1)	Inhibiting angiogenesis and myocardial regeneration after the myocardial infarction Facilitating ferroptosis and aggravating acute myocardial infarction.	[147, 148, 149, 150, 155]
Cancer	Tumor angiogenesis	Upregulated	Angiogenesis genes (VEGF, FGF, ANGP2, VEGFR, FGFR2, EGFR, NRP2) Antioxidant genes (HO‐1)	The majority of studies showed that high BACH1 expression increases tumor angiogenesis only one report showed that BACH1 decreases the protein levels of HO‐1, p‐AKT, p‐ERK, eNOS, HIF1A, and VEGF, and inhibits angiogenesis in pancreatic cancer cells	[54, 218, 219, 220]
	Tumor metastasis	Upregulated	EMT genes (SNAIL, CXCR MMPs) Cell motility genes (IGF1R, PTK2) BMS genes (OPN, MMP‐1, CXCR4, FHL1, and DUSP1)	Facilitating the metastasis of breast cancer, non‐small cell lung cancer (NSCLC), pancreatic cancer, colorectal cancer, ovarian cancer, glioma, and renal cell carcinoma, by modulating EMT, ECM remodeling, and enhancing the expression of pro‐metastatic factors	[31, 39, 46, 50, 55, 74, 75, 78, 222, 223, 224, 225, 226, 227, 228, 229, 230, 231, 232, 233, 234, 235, 236, 237, 238, 239, 240, 241, 242, 243, 244, 245, 246, 247, 248, 249, 250, 251, 252, 253, 254, 255, 256, 257]
	Tumor metabolism	Upregulated	Metabolic genes (HK2, GADPH) ETC genes (UQCRC1, SLC25A15, ATP5D)	Enhancing glycolysis Inhibiting mitochondrial tricarboxylic acid (TCA) Inhibiting lactate‐mediated mitochondrial metabolism	[39, 42, 43, 62, 223, 260, 261, 262, 263]
	Tumor ferroptosis	Upregulated	Ferroptosis genes (SLC7A11, FTL, FTH1, SLC40A1, HO‐1)	Inhibiting the expression of key genes regulating ferroptosis pathways and intracellular labile iron metabolism, thereby promoting ferroptosis.	[15, 148, 242, 272, 273, 274, 275, 276, 277, 278, 279, 280, 281, 282, 283, 284, 285, 286, 287, 288, 289, 290]
	Tumor micro‐environment	Upregulated	APOBEC3A (A3A), Wnt1, immune checkpoints (ICs) genes, M2 TAMs markers, PDLIM2	Inhibiting the innate and acquired immune systems in various cancers, facilitating M2 phenotype switch of macrophages and the development of immunosuppressive tumor microenvironment, ultimately fostering cancer progression	[179, 295, 296, 297, 298, 299, 300, 301, 304, 305, 306, 307, 308, 309, 310]
Gastrointestinal Disorders	Viral hepatitis	Upregulated	BACH1/HO‐1 signaling miR‐200a‐3p/MAP3K2 axis	Decreased BACH1 mitigates the cytotoxic effects of HCV proteins Inhibiting CircBACH1 impedes HBV replication and hepatocellular carcinoma progression	[305, 313, 314, 315, 316, 317, 318, 319, 320, 321]
	NAFLD	Upregulated	Insulin signaling genes	Increased in individuals with obesity and patients with NAFLD	[87, 323, 324]
	Hepatic injury	Upregulated	Antioxidant genes	Implicated in liver damage caused by a variety of injurious stimuli, such as anti‐tuberculosis drug‐induced hepatotoxicity (ATDH), aryl hydrocarbon receptor (AhR)‐mediated hepatotoxicity, LPS and Sepsis‐induced liver injury, acute CCl4, Aflatoxin B1 (AFB1), inorganic arsenic‐induced liver damage, APAP‐induced liver injury, and ischemia‐reperfusion caused liver injury	[60, 325, 326, 327, 328, 329, 330, 331, 332, 333, 334, 335, 336, 337, 338, 339, 340]
	Inflammatory bowel disease (IBD)	Upregulated	Inflammatory‐ related genes (CXCL9, TIMP1, PTGS2 CXCL1, MMP9, IL1B, CXCL8, IL6) Antioxidant genes (HO‐1)	Regulating genes linked to the Mayo score in ulcerative colitis BACH1 deficiency relieves various colitis through upregulation of HO‐1 and M2 polarization anti‐inflammatory response in macrophages	[85, 180, 345, 346, 347, 348, 349]
	Intestinal damage	Upregulated	NF‐κB signaling Adhesion molecules	BACH1 inhibition protects individuals from different types of intestinal damage such as NSAID‐associated enteropathy, domethacin‐induced intestinal injury, and intestinal ischemia‐reperfusion injury	[351, 352, 353]
Neurodegenera‐tive Disease	Down syndrome with Alzheimer's disease	Upregulated	HO‐1/BVR‐A pathway Microtubule‐associated protein tau (MAPT)	Trisomy of BACH1 inhibits antioxidant genes HO‐1, leading to inhibition of HO‐1/BVR‐A pathway and facilitating AD pathology progression in DS individuals BACH1 suppress MAPT expression, leading to the observed molecular gender differences in tauopathies and AD	[357, 358, 359, 360, 361, 362, 363, 364, 365, 366, 367, 368, 369]
	Parkinson's disease	Upregulated	Oxidative stress genes Non‐ARE genes (related to neuronal cell survival)	Ablation of BACH1 reduces MPTP‐induced oxidative stress and neuroinflammation Deletion of BACH1 stimulates ARE‐mediated neuroprotective pathways, and upregulation of many Non‐ARE genes, which related to neuronal cell survival and beneficial for Parkinson's patients.	[375, 376]
	Multiple sclerosis	Upregulated	HO‐1 Immune genes (MHC‐I genes H2‐Q4 and H2‐Q7, and MHC‐II pathway gene Cd74)	Deficiency of BACH1 in mice with EAE, results in less inflammation in the central nervous system (CNS) through the HO‐1‐dependent mechanism. A candidate gene for MS regulation, targeting the immune regulation of oligodendrocytes	[380, 381, 382, 383]
	Cerebral stroke	Upregulated	Inflammation & apoptosis ‐associated genes, COX2	BACH1 drives the ischemic core‐associated microglia (ICAM) production, which induce excessive neuroinflammatory responses and aggravate brain damage. BACH1 promotes ferroptosis via actuating KDM4C‐induced COX2 demethylation under cerebral ischemia‐reperfusion injury	[391, 392, 393, 394, 395, 396, 397, 398, 399, 400]
Neurodegenera‐tive Disease	Injurious stimulus	Upregulated	OXPHOS related genes HO‐1	Reduced BACH1 relieves MB‐mediated neurotoxicity, synapse formation induced by Pb, spinal cord injury, and oxygen‐glucose deprivation/reoxygenation‐induced neuronal injury BACH1 modifies microglial metabolism and microglial BACH1‐deficient mice display neuronal differentiation abnormalities and anxiety‐like behaviors	[403, 404, 405, 406, 407]
Diabetes	Dysfunction of pancreatic β‐cells and hepatic insulin resistance	Upregulated	Metabolic and oxidative stress genes Insulin signaling	Modulating the metabolic and oxidative stress responses to palmitate, contributing to the dysfunction of human pancreatic β‐cells Hepatocyte‐specific BACH1 deficiency elevates insulin signaling and alleviates abnormal glucose homeostasis regulation in high‐fat diet (HFD)‐caused hepatic insulin resistance, and finally prevents individuals from HFD‐induced steatosis	[23, 94, 195, 410]
	Diabetic cardiovascular complications	Upregulated	AKT3	Accelerating hyperglycaemia‐induced EndMT via facilitating of AKT3 transcription, promoting diabetic cardiovascular complications	[411, 412]
	Diabetic nephropathy	Upregulated	Fibrosis related genes Inflammation related genes	Acting as a core transcription factor of injured proximal tubule (PT) cells, promoting diabetic nephropathy (DN)	[412, 413, 414, 415, 416]
Respiratory Diseases	Pulmonary fibrosis	Upregulated	Oxidative stress genes ERK signaling TGFβ/SMAD signaling	BACH1 suppression relieves fibrosis and inflammation by blocking the ERK signaling. BACH1 silence impedes oxidative stress and BLM‐inducing the M2 macrophage phenotype through activating the TGFβ/SMAD signaling to alleviates pulmonary fibrosis.	[90, 422, 423, 424, 425]
Hematological disorders	Anemia	Upregulated	Iron cycling genes (SPIC, SLC40A1, HO1)	For β‐thalassemia patients, reduced BACH1 expression leads to enhanced erythrophagocytosis and iron‐recycling For Sickle cell disease (SCD), BACH1 inhibition acts as a therapy	[426, 427, 428, 429, 430, 431, 432, 433, 434, 435, 436, 437, 438, 439, 440, 441, 442, 443]
Hematological disorders	Leukemia/ multiple myeloma	Upregulated/ Downregulated	Antioxidant genes (HO‐1)	Inhibiting the survival of AML cells, suppressing AML progression Playing a beneficial role in MM	[444, 445, 446, 447, 448, 449, 450]
Skin damage		Upregulated	Antioxidant genes (HO‐1) Ferroptosis related genes	BACH1 inhibition protects against ultraviolet A(UVA)‐irradiation‐induced damage to skin keratinocytes and fibroblasts Suppressing BACH1‐related ferroptosis, δ‐Tocotrienol preconditioning boosts the potential of bone marrow‐derived mesenchymal stem cells in wound healing promotion	[95, 451, 452, 453, 454, 455, 456, 457, 458, 459, 460, 461, 462, 463, 464]

## 9 BACH1 and Hematological Disorders

### 9.1 BACH1 and Anemia

BACH1 has a close connection with the genesis and hematological disorders. Anemia is a prevalent disease in the hematological system, among which iron deficiency anemia occurs most commonly.^[^ ^426^, ^427^ ^]^ BACH1 deficiency causes more severe anemia and a delayed recovery in mice with low iron conditions. BACH1 equilibrates the levels of globin and heme through repressing globin and HO‐1 in erythroblasts upon iron lacking, Accurately adapting erythroblasts to iron deficiency and preventing toxic accumulation of non‐heme globin are dependent on the iron‐heme‐BACH1 axis.^[^ ^428^ ^]^ Hemolysis occurs when red blood cells are destroyed, and macrophages phagocytose damaged red blood cells to prevent the extracellular release of toxic hemoglobin and heme, transforming into erythrophagocytes.^[^ ^429^ ^]^ A regulatory network including BACH1 coordinates the unique transformation of phenotypes, which negatively regulates inflammation and immunity, as well as the disease‐specific outcomes of hemolytic anemia.^[^ ^430^ ^]^ Thalassemias are anemia caused by the mutations in genes encoding the chains of hemoglobin. Severe thalassemia is linked to iron overload and oxidative damage to tissues, leading to cardiovascular complications.^[^ ^431^ ^]^ Vascular endothelial dysfunction and increased arterial stiffness also exist in pediatric thalassemia patients, with the upregulation of BACH1 expression and downregulation of HO‐1 serving as an adaptive antioxidant gene response.^[^ ^432^ ^]^ In a group of 47 β‐thalassemia patients from Malaysia, BACH1 expression positively correlated with age, levels of α‐ and β‐globin chains, and HO‐1 protein, while negatively correlated with reticulocyte level, suggesting a compensatory response to restore globin balance and reduce oxidative stress in β‐thalassemia patients.^[^ ^433^, ^434^ ^]^ β‐thalassemia patients suffer from secondary iron overload due to enhanced gastrointestinal iron absorption,^[^ ^435^ ^]^ and the accumulation of excessive unmatched α‐globin chains causes premature red blood cells (RBC) hemolysis, which increases iron turnover.^[^ ^436^ ^]^ For β‐thalassemia patients, the reduced expression of the BACH1 gene leads to enhanced erythrophagocytosis and iron‐recycling by facilitating CD163, which is involved in the clearance of free plasma hemoglobin, and iron cycling genes (SPIC, SLC40A1, and HO1).^[^ ^437^ ^]^ Similarly, another study also indicated that the promoted miR‐155 expression in activated monocytes results in elevated phagocytic activity by inhibiting BACH1 in β‐thalassemia.^[^ ^438^, ^439^ ^]^ Sickle cell disease (SCD) is initiated as consequence of a point mutation in the β‐globin chain of hemoglobin, which leads to a substitution of valine for glutamate at position 6, manifesting as chronic hemolytic anemia.^[^ ^440^ ^]^ BACH1 exerts a significant function to SCD pathogenesis, and some small molecule drugs (hydroxyurea, ASP8731, and HPP‐D) can treat SCD via BACH1 inhibition.^[^ ^441^, ^442^, ^443^ ^]^ Hence, targeting BACH1 can reduce iron overload and oxidative stress, improving the treatment of anemia, thalassemia, sickle cell disease, and related disorders.

### 9.2 BACH1 and Leukemia or Multiple Myeloma

Leukemia is a disease caused via hematopoietic stem cells malignancy, resulting in the abnormal proliferation and differentiation of white blood cells. Acute leukemia has an acute onset and rapid progression, mainly including acute lymphoblastic leukemia (ALL) and acute myeloid leukemia (AML).^[^ ^444^, ^445^ ^]^ Researchers develop an integrated approach for gene expression profiling to uncover better predictors of clinical behavior in ALL, and reveal BACH1 as ALL low‐risk markers.^[^ ^446^ ^]^ It seems that BACH1 plays the role of a protector in leukemia. BACH1 inhibits the survival of AML cells by downregulating HO‐1.^[^ ^447^ ^]^ FBXO22 promotes AML progression by degrading BACH1, and overexpression of BACH1 suppresses AML progression.^[^ ^448^ ^]^ Bortezomib (Btz), a proteasome inhibitor, is clinically effective in multiple myeloma (MM) but ineffective in AML. The mechanism of drug resistance is that it rapidly inactivates BACH1, enabling NRF2‐regulated cytoprotective and detoxification genes to be induced rapidly, thus preventing AML cells from bortezomib‐mediated apoptosis.^[^ ^449^ ^]^ BACH1 plays a beneficial role in MM as well. BACH1 is upregulated in MM patients, and those in the high‐expression BACH1 group have a better prognosis after Btz‐treatment.^[^ ^450^ ^]^ Therefore, functionally upregulating BACH1 is a potential anti‐leukemia and MM treatment strategy.

## 10 BACH1 and Skin Health

The skin is constantly in contact with environmental factors, and exposure to adverse conditions often causes oxidative stress.^[^ ^451^, ^452^ ^]^ Epidermal differentiation is vital for maintaining normal skin structure and function with keratinocytes, as the epidermis’ main cell type.^[^ ^453^ ^]^ HO‐1 is typically found in the upper epidermis of normal skin, with the highest expression level in the granular layer, and its expression is also linked to the differentiation of keratinocytes.^[^ ^454^ ^]^ There has been evidence in previous studies that BACH1, which inhibits HO‐1 expression and is important in oxidative stress, functions crucially in the normal function and differentiation of keratinocytes.^[^ ^451^, ^455^ ^]^ The inhibition of BACH1 protects against ultraviolet A(UVA)‐irradiation‐induced damage to skin keratinocytes and fibroblasts.^[^ ^95^, ^456^, ^457^ ^]^ UVA induces the antioxidant effect of HO‐1, but excessive HO‐1 expression is detrimental. UV also promotes the expression of BACH1 to inhibit the overexpression of HO1, which is a compensatory mechanism of the body.^[^ ^456^ ^]^ It has been shown that various chemical reagents can offer protection to keratinocytes against injury stimuli by targeting BACH1. Acetyl‐11‐keto‐β‐boswellic acid (AKBA) is a vital constituent of the gum resin of the medicinal plant Boswellia serrata (Roxb. ex Colebr), and it is utilized to treat inflammatory disorders like rheumatoid arthritis and inflammatory bowel disease.^[^ ^458^, ^459^ ^]^ By inhibbiting BACH1 and Reducing ROS production, AKBA protects skin cells from UVA‐mediated damage.^[^ ^460^ ^]^ Topical treatment of green tea polyphenols (GTPs) emulsified in carboxymethyl cellulose (CMC‐Na), which evaluating the stabilizing effect of GTPs, can protect against acute ultraviolet light B (UVB)‐mediated photodamage in hairless mice through enhancing the nuclear export of BACH1.^[^ ^461^ ^]^ Phenylpropanoid glycosides (PPGs) derived from plant cell cultures exert an inducing effect on HO‐1 gene expression in human keratinocytes via influencing the equilibrium of NRF2 and BACH1.^[^ ^462^ ^]^ Cannabidiol (CBD), a prominent non‐psychotropic phytocannabinoid, triggers antioxidant pathways in keratinocytes through the specific targeting of BACH1.^[^ ^463^ ^]^ Additionally, by suppressing BACH1‐related ferroptosis, δ‐Tocotrienol preconditioning boosts the wound healing capabilities of bone marrow‐derived mesenchymal stem cells.^[^ ^464^ ^]^ Thus, future research may further reveal its potential applications in skin diseases, particularly in anti‐aging, protection against ultraviolet damage, and promoting skin repair.

## 11 Manipulate the BACH1/NRF2 Signaling

### 11.1 NRF2 Activators

Nuclear factor erythroid 2‐related factor 2 (NRF2) and BACH1, both members of the Cap “n” Collar family, attach to similar DNA sequences, specifically the MAREs region.^[^ ^276^, ^465^ ^]^ In cells with high BACH1 level, BACH1 inhibits NRF2 from binding to the MAREs region in the promoter of NRF2 target genes^[^ ^466^, ^467^ ^]^ NRF2 regulates hundreds of target genes transcription, involved in antioxidant genes for detoxifying xenobiotics and neutralizing reactive oxygen species (ROS),^[^ ^272^ ^]^ immunomodulation and energy metabolism‐related genes.^[^ ^376^ ^]^ Normally, the NRF2 protein undergoes polyubiquitination in the Cullin lII E3‐ubiquitin ligase complex and binds to the dimeric kelch‐like ECH‐associated protein 1 (KEAP1), leading to its breakdown through proteasomal degradation.^[^ ^468^ ^]^ However, oxidative stress modifies the cysteine residues of KEAP1, resulting in its inactivation under oxidative stress. Consequently, NRF2 protein separates from KEAP1 and transfers into the nucleus.^[^ ^468^, ^469^, ^470^ ^]^ NRF2 dimerizes with MAF proteins and binds to MARE in the promoter region of the target genes and triggers gene transcription. Meanwhile, BACH1 is inactivated and dissociates from DNA under oxidative stress.^[^ ^83^ ^]^ BACH1 antagonizes the actions of NRF2, leading to increased ROS production in cancer and neurodegenerative diseases, suppression of angiogenesis in ischemic cardiovascular disease, and facilitation of cellular senescence and apoptosis in many diseases. Indeed, with the increase of age, BACH1 expression rises, while NRF2 expression drops, which regulates cellular cytoprotective responses.^[^ ^471^, ^472^, ^473^ ^]^ Meanwhile, lifestyle diseases are commonly associated with downregulation of the NRF2 pathway. Therefore, NRF2 activation is crucial to alleviating the harmful effects of inflammation and oxidative stress for these patients.^[^ ^88^, ^94^, ^472^, ^474^, ^475^ ^]^ Keap1, the most validated target for NRF2 activation, is a redox sensor containing several cysteine residues, whose oxidation or modification compromises either dimeric structure formation or combination to ubiquitin ligase, resulting in NRF2 protein release and nuclear import.^[^ ^476^, ^477^ ^]^ The Food and Drug Administration (FDA)‐approved NRF2 activators, Tecfidera and Skyclarys, are non‐specific alkylating agents targeting Keap1, used for treating patients with multiple sclerosis and Friedreich's ataxia. Nevertheless, Tecfidera's trials showed side effects like leucopenia and significant gastrointestinal problems, while Omaveloxolone has very common side effects, over 10%, including elevated liver enzymes (AST/ALT), headache, etc.^[^ ^476^ ^]^ A number of NRF2 activators also possess herbal medicines functions through alkylation/covalent modification, such as sulforaphane (broccoli), curcumin (turmeric), nordihydroguaiaretic acid (chapparal), flavonoids (quercetin, fisetin).^[^ ^478^ ^]^ However, many natural NRF2 agonists are unstable under physiological conditions, with extremely low bioavailability,^[^ ^479^ ^]^ and their reactions with cysteine residues are often irreversible, which can cause abnormal and persistent activation of NRF2 and may lead to carcinogenesis,^[^ ^480^ ^]^ severely limiting their clinical application. Actually, the NRF2‐actuated program has a feedback regulation via the transcriptional suppressor BACH1, which is an NRF2 target gene and represses the transcription of many NRF2‐dependent and ‐independent genes.^[^ ^376^, ^481^ ^]^ Moreover, the majority of NRF2‐based pharmacophores (e.g., curcumin, fumarate, sulforaphane, triterpenoids, etc.) activate NRF2 by enhancing oxidative stress and are detrimental chemicals.^[^ ^478^ ^]^ The increase of oxidative load on cells that are already unhealthy may strikingly counteract the advantages of activating the antioxidant genetic program, rendering it challenging to clearly interpret the post‐treatment outcomes.^[^ ^478^ ^]^ Especially in the MPTP mouse model of Parkinson's disease, only the pretreatment with NRF2 activators provides protection, not the post‐treatment.^[^ ^478^ ^]^ Hence, an alternative strategy is to bypass the Keap1‐ NRF2 interaction via repressing BACH1.

### 11.2 BACH1 Inhibitors

The development of small molecule inhibitors targeting BACH1 for potential therapeutic applications has been a major focus in recent years (**Table** **3**). Hemin is a suppressor of BACH1 DNA‐binding activity and induces BACH1's nuclear export and degradation,^[^ ^23^, ^27^, ^35^, ^482^, ^483^ ^]^ which can reverse the inhibition of VEGF activity in human microvascular endothelial cells (HMEC‐1) cells caused by hyperoxia by inhibiting BACH1.^[^ ^119^ ^]^ Research has indicated that augmenting heme synthesis by externally supplementing the heme precursor 5‐aminolevulinic acid (ALA) can reduce cancer cell growth and improve kidney health by destabilizing BACH1.^[^ ^484^, ^485^ ^]^ Intravenous hemin (Panhematin) has been approved by the Food and Drug Administration as acute porphyria therapy.^[^ ^486^ ^]^ Metalloporphyrins with metals like zinc or tin, other than iron, do not catalyze ROS production and act as canonical inhibitors of BACH1 by binding to BACH1's CP motifs.^[^ ^487^, ^488^, ^489^ ^]^ HPP‐4382, a non‐electrophilic compound, impairs the DNA binding ability of BACH1 through imitating heme, yet seems not to have an impact on the expression and nuclear distribution of BACH1.^[^ ^490^ ^]^ A new oral derivative of HPP‐4382, namely HPP‐D, activates γ‐globin by competitively inhibiting the binding of BACH1 to antioxidant response elements and promoting NRF2 combination in sickle erythroid progenitors.^[^ ^443^ ^]^ In a Parkinson's disease mouse model, a substituted benzimidazole (HPPE) aids in releasing BACH1 from DNA binding elements and translocation out of the nucleus, and as an effective and safer analog of heme, it is an underlying therapy for Parkinson's disease.^[^ ^376^ ^]^ ASP8731, formerly referred to as ML‐0207, is an alternative small molecule suppressor of BACH1 inhibiting inflammation and vaso‐occlusion and inducing fetal hemoglobin in sickle cell disease.^[^ ^376^, ^442^ ^]^ A small molecule 1‐Piperazineethanol, α‐[(1,3‐benzodioxol‐5‐yloxy) methyl] ‐4‐(2‐methoxyphenyl) (M2) is found to be the inhibitor of BACH1. M2 as well as its analogues inhibited AFB1‐induced cell death and improved weight loss and liver injury.^[^ ^334^ ^]^ The synthetic acetylenic tricyclic bis (cyanoenone), TBE31, is a powerful activator of NRF2 absent of BACH1 activity.^[^ ^491^ ^]^ TBE56, a biotinylated form of TBE31, boosts the degradation of BACH1 through a mechanism including the E3 ligase FBXO22. TBE56 is a sustained BACH1 inhibitor, more powerful than hemin by 50‐fold. TBE56 degrades BACH1 in lung and breast cancer cells, and impairs breast cancer cell migration and invasion, while TBE31 has no valid effect.^[^ ^492^ ^]^ The synthetic oleanane triterpenoid 2‐cyano‐3,12‐dioxooleana‐1,9(11)‐dien‐28‐oic acid (CDDO) serves as an activator of NRF2. While both CDDO and its derivatives, namely CDDO‐trifluoromethyl‐amide (CDDO‐TFEA) and CDDO‐Bardoxolone‐Methyl (CDDO‐Me), activate NRF2 in a similar way, only CDDO‐Me and CDDO‐TFEA suppress BACH1 through reducing its nuclear level, making them dual KEAP1/BACH1 inhibitors impeding the invasion of lung cancer cells.^[^ ^491^ ^]^

**Table 3:** Mechanism and function of BACH1 inhibitors.

BACH1 inhibitor	Mechanism	Function/Treatment/Defect	References
Hemin	Suppressing BACH1 DNA‐Binding activity and inducing BACH1's nuclear export and degradation	Intravenous hemin (Panhematin®) is an acute porphyria therapy Improves retinal angiogenesis by reversing hyperoxia‐induced VEGF inhibition Cytotoxic effect	[23, 27, 35, 119, 482, 483, 486]
5‐aminolevulinic acid (ALA)	Heme precursor, augmenting heme synthesis	Reduce cancer cell growth and improve kidney health by destabilizing BACH1	[484, 485]
Metalloporphyrins	Binding to BACH1's CP motifs	Act as canonical inhibitors of BACH1 Poorly penetrating membranes and inhibiting HO‐1 activity	[487, 488, 489]
HPP‐4382	A non‐electrophilic compound impairing the DNA binding ability of BACH1 through imitating heme	Potential anti‐leukemia treatment	[490]
HPP‐D	Activates γ‐globin by competitively inhibiting the binding of BACH1 to antioxidant response elements and promoting NRF2 combination	Potential treatment for sickle cell disease	[443]
HPPE	Releasing BACH1 from DNA binding elements and translocation out of nucleus	Effective and safer analog of heme Underlying therapy for Parkinson's disease	[376]
ASP8731 (ML‐0207)	Small molecule suppressor of BACH1	Inhibiting inflammation and vasoocclusion Inducing fetal hemoglobin in sickle cell disease	[376, 442]
M2	Small molecule suppressor of BACH1	Inhibited AFB1‐induced cell death Improved the symptoms of weight loss and liver injury	[334]
TBE56	Promoting BACH1 degradation through E3 ligase FBXO22, more powerful than hemin by 50‐fold	Degrading BACH1 in lung and breast cancer cells, impairing breast cancer cell migration and invasion	[492]
CDDO and the derivatives (CDDO‐TFEA, CDDO‐Me)	Activating NRF2 *only CDDO‐Me and CDDO‐TFEA suppress BACH1 through reducing its nuclear levels	CDDO‐Me and CDDO‐TFEA are dual KEAP1/BACH1 inhibitors impeding the invasion of lung cancer cells	[491]
Cannabidiol (CBD)	Mediating BACH1 nuclear export and cytoplasmic proteasomal degradation	Inducing some NRF2 target genes expression in a NRF2 independent way in keratinocytes Treatment of various skin diseases	[463]
Isomeric O‐methyl cannabidiol quinone	Cannabidiol derivative, functions similar to CBD	Activating NRF2 in cell‐culture models of neurodegenerative diseases	[493]
Hyperoside (HYP)	Facilitating Crm1‐dependent BACH1 nuclear export	Protective role in oxidative damage in hepatocytes and CCl4‐mediated acute liver injury	[333]
Hyperforin (HPF)	Suppress BACH1 expression	Enhancing HO‐1's expression Triggering lipid peroxidation in melanoma cells and hindering metastasis	[494]
Rapanone and Nectandrin B	Binding to BACH1 allosteric region	Regulate the expression of downstream target genes related to cancer progression particularly in metastasis	[495]
Sinensetin (SIN)	Enhancing BACH1 ubiquitination degradation	Promoting HO‐1 expression, suppressing oxidative stress in periodontal ligament cells A potential drug for periodontitis treatment	[496]
Sophoricoside (SOP)	Suppressing BACH1 expression	Improves methicillin‐resistant Staphylococcus aureus‐induced acute lung injury	[497]
S‐1‐propenylcysteine (S1PC)	Promoting the degradation of BACH1	A foundation for the antioxidant properties Enhances endothelial function	[498, 499]
Yeast hydrolysates (YH)	Suppressing BACH1 expression	YH supplementation improved antioxidant capacity and innate immunity of blunt snout bream hepatocytes.	[500]
Cottonseed protein meal hydrolysates (CPH)	Suppressing BACH1 expression	CPH enhanced hepatocyte metabolism, as well as improved antioxidant capacities and innate immunity of blunt snout bream hepatocytes.	[501]
Rosuvastatin	Suppressing BACH1 expression by increasing microRNA let‐7a	Reduced BACH1 expression in the vascular endothelium of hyperlipidemic mice alleviates vascular inflammation. Atheroprotective effects on endothelial cells	[14]
Remifentanil (RE)	Inhibiting the expression of BACH1	Suppressing NF‐κB signaling pathway by decreasing TRAF3 in a rat model of hypoxic‐ischemic brain damage (HIBD) Mitigating HIBD‐caused cognitive impairment Ameliorating hepatic ischemia‐reperfusion injury (HIRI)	[502, 503]

Recently, the identification of BACH1 inhibitors in natural products provides an alternative approach to drug discovery. Cannabidiol (CBD), a non‐psychotropic phytocannabinoid derived from the cannabis plant, promotes the nuclear export and cytoplasmic proteasomal degradation of BACH1 for the treatment of various skin diseases.^[^ ^463^ ^]^ The novel cannabidiol derivative, isomeric O‐methyl cannabidiol quinone, exhibits similar impacts on BACH1 and triggers NRF2 in cell‐culture models of neurodegenerative diseases.^[^ ^493^ ^]^ Hyperoside (HYP), a naturally occurring flavonoid present in fruits and vegetables, and its’ protective role in oxidative damage in hepatocytes and CCl4‐mediated acute liver injury include Crm1‐dependent BACH1 nuclear export.^[^ ^333^ ^]^ Hyperforin (HPF), an acylphloroglucinol compound discovered in large quantities in Hypericum perforatum extract, enhances HO‐1′s expression by suppressing BACH1, triggering lipid peroxidation in melanoma cells and hindering metastasis.^[^ ^494^ ^]^ Rapanone and Nectandrin B, derived naturally from rosewood and nutmeg respectively, can bind to BACH1 allosteric region to modulate the expression of downstream target genes that are associated with cancer progression particularly in metastasis.^[^ ^495^ ^]^ Sinensetin (SIN), a nature polymethoxylated flavonoid, induces the reduction of BACH1 and serve as a potential therapeutic drug for periodontitis by enhancing the BACH1 ubiquitination degradation, promoting the expression of HO‐1, and suppressing oxidative stress in periodontal ligament cells.^[^ ^496^ ^]^ Sophoricoside (SOP), an isoflavone glycoside found in the fruit of Sophora japonica l., improves methicillin‐resistant Staphylococcus aureus‐induced acute lung injury via inhibiting BACH1.^[^ ^497^ ^]^ S‐1‐propenylcysteine (S1 PC), a compound found in aged garlic extract (AGE), serves as a foundation for the antioxidant properties and has been demonstrated to enhance endothelial function by promoting the degradation of BACH1.^[^ ^498^, ^499^ ^]^ Certain protein hydrolysates, such as yeast hydrolysates (YH) and cottonseed protein meal hydrolysates (CPH), exhibit antioxidant properties by inhibiting BACH1.^[^ ^500^, ^501^ ^]^

Our study finds that Statins, especially Rosuvastatin, represses the expression BACH1 via increasing microRNA let‐7a in ECs. Reduced BACH1 expression in the vascular endothelium of hyperlipidemic mice ultimately alleviates vascular inflammation.^[^ ^14^ ^]^ In addition, the anesthesia drug Remifentanil (RE) inhibits the expression of BACH1, which subsequently suppresses the NF‐κB signaling pathway by decreasing TNF receptor‐associated factor 3 (TRAF3) in a rat model of hypoxic‐ischemic brain damage (HIBD), and mitigates cognitive impairment resulting from HIBD.^[^ ^502^ ^]^ A separate study demonstrated that RE decreased oxidative stress and inflammation by suppressing BACH1, thereby ameliorating hepatic ischemia‐reperfusion injury (HIRI).^[^ ^503^ ^]^ The inhibition of BACH1 by clinical medications offers a novel approach treating the relevant diseases. It is necessary to further study the specific mechanisms by which these drugs inhibit BACH1 in the future, as well as their potential applications in clinical therapy. Moreover, the development of more specific and effective BACH1 inhibitors is crucial to provide more efficacious approaches for the treatment of diseases.

## 12 Conclusions and Future Perspectives

BACH1 is associated with oxidative stress responses, and it regulates the antioxidant defense system by controlling the expression of genes associated with ROS metabolism. BACH1 influences the cellular response to oxidative stress by balancing oxidants and antioxidants. BACH1 effectively regulates multiple cellular processes like cell growth, differentiation, senescence, and apoptosis. In most cases, it functions by binding to certain DNA sequences and modulating the expression of target genes. However, in hESCs, BACH1 regulates the pluripotent gene expression independently of its transcriptional factor function. It enhances the protein stability of pluripotency factors by binding to pluripotency factors and recruiting the deubiquitinating enzyme USP7, thus preserving stem cells' self‐renewal ability. Lineage‐specific differentiation of tissue cells from stem cells is essential for regenerative medicine, especially for cardiovascular system diseases, as the lack of cardiovascular cell regeneration after injury can lead to heart failure, seriously threatening people's health. BACH1 promotes the differentiation of VSMCs after mesoderm induction by recruitment of CARM1. In the future, the role and mechanism of BACH1 in stem cell differentiation into vascular endothelial and myocardial cells can be studied to provide a new therapeutic strategy for cardiovascular regenerative medicine.

**Figure 10:**
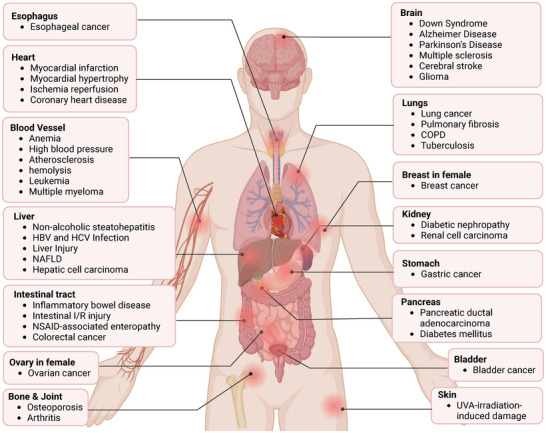
BACH1 and diseases. BACH1 has significant functions in diverse pathological states, including cardiovascular system disorders, tumor growth and metastasis, neurodegenerative diseases, gastrointestinal problems, hematological disorders skin health, etc. The illustration was generated using BioRender under a licensed subscription (Agreement number: EE27P3KQ6U).

BACH1 also exerts significant roles in diverse pathological conditions, including cardiovascular system diseases, tumor growth and metastasis, neurodegenerative diseases, gastrointestinal disorders, leukemia, pulmonary fibrosis, and skin diseases (**Figure** **10** and Table 2). BACH1 regulates metabolism‐related genes including adipocyte function, glycolysis, and oxidative phosphorylation as well. Our research found that BACH1 lowers the sensitivity of the insulin signaling pathway, indicating that BACH1 also takes a crucial part in the development of metabolic disorders. Glucose and lipid metabolism disorders are also key factors affecting the development of cardiovascular diseases, and more exploration is needed for the role and mechanism of BACH1 in metabolic cardiovascular diseases. In most cases, BACH1 aggravates the progression of the disease, and inhibition of BACH1 is beneficial for the majority of diseases. BACH1 knockout ameliorates cardiovascular diseases like atherosclerosis, restenosis, and pathological cardiac hypertrophy. Inhibition of BACH1 has therapeutic effects on inhibiting the growth and metastasis of tumors, neurodegenerative diseases, β‐thalassemia, Sickle cell disease, pulmonary fibrosis, skin diseases, etc. Therefore, the development of inhibitors targeting BACH1 has good application prospects. In the future, based on different cell types and disease states, the design of precise targeting therapeutic regimens against BACH1 has potential clinical application value.

## Conflict of Interest

The authors declare no conflict of interest.

## Author Contributions

X.Wei and D.M. conceived the concept for this comprehensive review and wrote the draft of the manuscript. X. Wei, Y.H., S.T., and R.L. made the figures and tables. Y.Y. did the literature search and review. J.G., Q.J., X.Z., and X.W. provided valuable comments. All authors have read and approved the article.
